# Passive digital health technologies for Alzheimer’s disease screening and diagnosis: a systematic review

**DOI:** 10.1038/s41746-026-02650-1

**Published:** 2026-04-25

**Authors:** Igor Matias, Paweł Prociów, Eric J. Daza, Matthias Kliegel, Katarzyna Wac

**Affiliations:** 1 Quality of Life Technologies Lab, University of Geneva, Geneva, Switzerland; 2 Cognitive Aging Lab, University of Geneva, Geneva, Switzerland; 3 DSW University of Lower Silesia, Wrocław, Poland; 4 Stats-of-1, Menlo Park, California United States of America; 5 Boehringer Ingelheim Pharmaceuticals Inc., Ridgefield, California United States of America

**Keywords:** Biomarkers, Diseases, Health care, Medical research

## Abstract

Passive digital health technologies (DHTs) are increasingly promoted as scalable tools for detecting Alzheimer’s disease and related dementias (ADRD) earlier than routine clinic visits. We searched six major databases for English-language studies published between January 2014 and July 2024 that used passively collected, real-world DHT data for ADRD screening or diagnosis. Thirty studies met the criteria. Population sizes were highly skewed (median = 87; range 12-82,829), and most designs were longitudinal (53%) and fully passive (68%). Wrist-worn accelerometers and photoplethysmography sensors dominated, though several studies also used gait, sleep, voice, radar, or posture-tracking devices. A cross-study synthesis showed that those two modalities were primarily applied to memory, attention, and language tasks. Nineteen studies reported median accuracy, sensitivity, specificity, and precision between 80-90%, with F1-score and AUC medians approaching 78%, though relying on in-sample cross-validation rather than external cohorts. Reference standards varied widely, data-quality criteria were seldom reported, and fewer than 5% shared datasets publicly. Classification was the predominant modeling strategy, with regression emerging only in recent years. Overall, passive DHTs show promise as low-burden triage tools for population-level ADRD screening, but routine deployment will require more diverse cohorts, harmonized reporting, multimodal privacy-preserving analytics, and rigorous human-factors evaluation.

## Introduction

Dementia is a term used to describe several diseases affecting memory, thinking, and the ability to perform daily activities^1^. It is estimated that almost 70 million people live with the condition worldwide, which is expected to double every 20 years^2^. Specifically, in Switzerland, around 157,000 people are affected by it, with more than 33,000 new cases reported yearly^3^. Overall, by 2050, 152 million individuals worldwide will live with dementia, an increase that will be more impactful in low- and middle-income countries^4^. Its leading cause, *Alzheimer’s disease* (AD), is described as a life-course disease in which the onset and the clinical symptom manifestation can be separated by decades^5^. With a cure not yet found^6^ (despite treatment options having recently been available^7^), growing evidence has indicated that subtle changes in cognition, sensory, and motor daily capabilities may precede AD’s manifestations by several years^8^.

With a growing research focus, early AD detection can play a crucial role in changing how the disease is seen. Early diagnosis allows/would allow *People with Dementia* (PwD) to understand their prognosis and trajectory better, state their wishes for care, and organize personal finances for future needs, as pointed out by Ford et al.^6^. Such early diagnosis is currently attempted using several tools, including (but not limited to) neuropsychological and cognitive tests^9^. However, these methods are often ineffective in detecting subtle deviations from the normal cognitive aging trajectory, a key point for recognizing the early stages of AD^10^. Additionally, current active assessment tools—that require active engagement from the patient, such as validated self-reports and cognitive tests—suffer from cultural and social desirability bias, practice effects, and ceiling effects, are time-consuming, and only provide episodic information^10^.

*Digital Health Technologies* (DHTs), including personal mobile and wearable sensors, are indicated as a possible way of shifting from active to passive monitoring of *AD and Related Dementias* (ADRD), allowing for continuous monitoring of several aspects of one’s daily life^5^. Those are ubiquitous and possible to use at a large scale^11^, with an increasing amount of research revealing a high association between passively collected DHTs and symptoms of neurodegenerative diseases^5,9^. In a landscape analysis, Lott et al.^12^ found that most current research employs commercially available DHTs, with the majority relying on ambient sensors, approaching clinical validation, and focusing primarily on overall cognition and specific types of memory. Contrary to active assessment tools, passive DHTs are ecologically valid^13^, allow for a fully remote and longitudinal approach^12^, which is crucial for early detection, when intervention is expected to have the most clinically meaningful impact. Additionally, these methods can also be used to enhance clinical trials (the current gold standard of research) by boosting recruitment, allowing for a more representative and diverse sample of the population^12^, and reducing within-participant noise^14^.

However, using DHTs in the search for passive ways to screen for or diagnose ADRD, seen as urgent by Kourtis et al.^10^, poses challenges that need to be addressed by researchers. DHTs must be accurate and applicable on a large scale; therefore, research should include multi-ethnic samples of the population, thereby increasing heterogeneity and replication^6,15,16^. Access to them must meet equity needs across countries and cultures, avoiding the magnification of existing inequalities in technology and health access^6^. Patients’ and clinicians’ concerns should be addressed, namely the intrusiveness of data being collected^5^, the security of data storage and usage^10,17^, the reliability of the devices with which DHTs are gathered^17^, and the overall understanding and trust in the output generated by such technologies^6^. Finally, multimodality and high frequency of DHT sampling must be explored. Popp et al.^5^ found potential in combining digital measures from multiple data streams over several years, which likely improves the prediction of cognitive impairment and early AD. High frequency was also proposed by Ford et al.^6^ and Kourtis et al.^10^, who defended that, for screening to be of the maximum value possible, it should be performed frequently and account for inter- and intra-subject variability over the lifespan.

Ultimately, passive DHTs applied to the screening and diagnosis of ADRD have the potential to change the paradigm of how early stages of disease and even disease onset forecasting are seen^5,9,12,17,18^. Importantly, within this manuscript, diagnosis refers to the classification tasks performed within research settings against defined reference standards, and not to independent clinical decision-making without comprehensive assessment. However, this remains an aspiration, as the research’s focus, funding, and understanding of technology opportunities and limitations fall short of the required level^5^.

In this article, we present the results and conclusions of a systematic literature review on the usage of passive DHTs for the screening and diagnosis of ADRD between 2014 and 2024. Its main contributions are:

- Comprehensive mapping of a decade of work: we screen 1608 to select 30 peer-reviewed studies published between 2014 and 2024, charting publication trends, venues, geographic origins, and study aims, and present the full PRISMA flow, search strings, and annex tables with the contents of the extraction sheet publicly available for reuse.
- Quantitative synthesis of DHTs’ choices and performance: we analyze the metrics reported in modeling papers and link them to specific input data, showing that multimodal devices consistently achieve high accuracy, sensitivity, and specificity (around 80–90%), with physical activity, sleep, and biological and physiological modalities leading to the most performing models.
- Critical appraisal of methodological and equity gaps: we analyze sample sizes, demographic spread, outcome definitions, data-quality practices, and data-sharing policies, exposing narrow cohorts, heterogeneous gold standards, and an almost 97% rate of closed datasets that together undermine reproducibility.
- Actionable agenda for future research and development: drawing on identified gaps, we outline concrete steps, harmonized reporting templates, FAIR data sharing, larger multi-ethnic cohorts, privacy-preserving multimodal analytics, and systematic user-acceptance testing, to guide the community toward safe, equitable, and clinically useful passive DHT screening and diagnosis of ADRD.

While prior reviews have examined DHTs in dementia research, their scopes differ substantially from ours. Some focus primarily on medical-grade or clinic-dependent assessments that cannot be deployed passively in daily life^19^, whereas others emphasize assistive or supportive technologies rather than tools intended for screening or diagnosis^20^. Additional reviews restrict themselves to a single modality—such as spatial navigation or gait—rather than synthesizing the full range of passive data streams^13^. Finally, several influential reviews focus mainly on remote but active assessments (e.g., self-reports, prompted cognitive tests), rather than continuous passive sensing^21^. Importantly, none has provided a systematic synthesis isolated to passive DHTs deployed in non-clinical, real-world settings specifically for screening or diagnosis. Our inclusion and exclusion criteria explicitly enforce this scope, distinguishing our review from broader analyses of digital biomarkers or general wearable-based monitoring. By narrowing the focus to technologies that operate unobtrusively in daily life, we consolidate a body of evidence not previously brought together—one that represents the most scalable, low-burden pathway toward population-level cognitive screening.

## Results

### Eligibility of studies

Given the complex definition of the inclusion and exclusion criteria used in this review, it is essential to clarify where these criteria were further defined for some of the included studies.

- Population’s cognitive state: we only reviewed studies in which research was done with data collected from *cognitively healthy* (CH) individuals or at any intermediate stage between CH and dementia caused by ADRD. However, other populations may have been included in the studies. For example, a study researching data from both AD and Parkinson’s disease patients would still be reviewed, as at least part of the data met our criterion.
- DHT usage: we reviewed only articles in which at least one of the goals was to employ a sensor collecting data passively in real-world settings. That is, some studies reviewed involved data collection that was active and required participant’s engagement but which was seen as feasible for a passive real-world application (e.g., using a portable EEG headband that requires the user to place it on their head, but for which a developed method can be used in scenarios in which the EEG is passively collected from other type of devices like earbuds). All of them reported analytical results directly relevant to cognitive screening or diagnosis. We did so in order not to focus solely on the research scenario, but instead to focus on real-world application cases.
- Study context or applicability of results: as for the technology usage, we decided to focus on the intended usage of the technology being researched. For this reason, some manuscripts reviewed may describe research performed with a device in a clinical setting, but only for validation or development purposes, while the actual application envisioned is passive and occurs outside the clinical facilities, i.e., in the participant’s or patient’s real-world environment.
- Research goal: this review aims to highlight recent research for screening or diagnosis of ADRD. Aligned with that, any article describing research focusing solely on creating or validating a tool to monitor ADRD patients after diagnosis was excluded. We did so because, although these systems can enable tracking of disease progression, they do not address the need for a ubiquitous and passive technology for scalable screening of ADRD in the healthy population.

In this review, we conceptualize screening as the broad umbrella that includes population-level identification of individuals who may warrant further evaluation. Within this framework, triage and risk stratification are considered operational components of screening, as they prioritize individuals based on estimated likelihood of impairment. In contrast, formal diagnosis refers to a comprehensive clinical determination based on established neuropsychological and/or biomarker criteria.

### Study quality evaluation

Across the 240 individual Newcastle–Ottawa scale (NOS)^22^ domain assessments (8 criteria × 30 studies), the two reviewer authors reached blinded agreement in 208 cases (86.7%), with remaining discrepancies resolved through discussion. Most of the studies (22, 73.3%,^23–44^) were evaluated following the case-control version of NOS, while the remaining followed the cohort form. The distribution of domain-level scores showed marked heterogeneity across studies (Fig. S1 in the Supplementary Materials). Most studies received three stars in the Selection domain, whereas Comparability scores were generally low, with nearly half of the studies scoring zero stars. Outcome-domain ratings were stronger overall, with the majority of studies receiving two stars.

Total NOS scores ranged from 1 to 9 stars (see Fig. S2 in the Supplementary Materials), averaged 6.0 ± 2.0 (6.4 ± 2.5 for cohort studies, 5.8 ± 1.8 for case-control). With a concentration in the mid-to-upper range, most studies (25, 83.3%) scored between five and nine stars, while only a small number fell below five stars (5, 16.7%^24,25,36,39,45^). A complete list of star ratings for each study, including Selection, Comparability, and Outcome domains, is provided in Table S1 in the Supplementary Materials.

### Metainformation RQ1: When, where, and by whom was the article published?

Considering the inclusion criteria specified that the articles selected must have been published later than 2014, it can be observed that since 2019, there is a notable increase in publications fitting the scope (Fig. S3 in the Supplementary Materials, with detailed information provided by Table S2 in the Supplementary Materials). This might reflect a recent growing interest or advancements in technological approaches to screening and diagnosing cognitive disorders, as well as technological improvements that make such studies more feasible.

The majority of reviewed research was published in peer-reviewed venues of the fields of computer science (10, 33.3%^23–25,30,31,37,44–47^), aging (four, 13.3%^26,28,29,41^), or medicine (four, 13.3%^27,42,43,48^), totaling 60.0% of the total 30 articles. Other fields were represented, including cognitive neuroscience (3 articles, 10.0%^34,49,50^), biomedical engineering, neurology, and public health (two articles each, 6.7%^35,51^^,^^38,39^^,^^33,52^, respectively), and multidisciplinary, neuroscience, and statistics (one manuscript each, 3.3%^32,34,36^, respectively). Figure S4 in the Supplementary Materials presents a graphical representation of that (Table S2 includes detailed information). The inclusion criteria of the review implied that the studies described must meet a certain standard. In this context, it is unsurprising that the majority of the selected articles were published in journals (23, 76.7%^26–29,32–36,38–44,46–52^) rather than conferences. The notable exception is publications in the field of computer science, which can be attributed to the disciplinary norms of the field, where top-tier conferences serve as primary outlets for the rapid dissemination of innovative technological developments, including algorithmic advancements and prototype evaluations, an important consideration in the fast-evolving domain of digital screening and diagnostics.

The quality of the reviewed publications, as assessed by journal quartile rankings and H-Index values, indicates a strong emphasis on high-impact research. A substantial proportion of the studies (16 out of 23, 69.6%^26,27,29,32–34,36,38,39,42,44,46,48–50,52^) were published in Q1 journals, representing the top 25% of journals within their respective fields. Additionally, the average H-Index of Q1 journals was 168.1 ± 95.2, notably higher than that of Q2 (82.0 ± 42.8) and Q3 (85.0 ± 39.6) journals. This distribution underscores the methodological and scientific robustness of the reviewed publications. The predominance of high-quartile journals enhances the reliability of the findings and provides a strong foundation for understanding the role of technology in facilitating the screening and diagnosis of cognitive disorders.

The geographic distribution of the reviewed studies reveals that most data collection took place in Europe and Asia, with these two continents accounting for the majority of publications (27, 90.0%, Europe^27–29,31,32,35,44,50,52^ Asia^24–26,30,33,34,37,38,40–43,45–49,51^, as in Fig. S5 and Table S3 in the Supplementary Materials). This trend is likely reflective of demographic realities, as many European and Asian countries, particularly Japan and South Korea, are among those most affected by rapidly aging populations^53^. These countries have well-documented aging demographics and developed healthcare and research infrastructures, which may explain their leading roles in studies focused on the technological screening and diagnosis of cognitive disorders, such as ADRD.

### Metainformation RQ2: What is the objective and scientific contribution?

The objectives and scientific contributions of the reviewed studies reflect the dominant theme of this review selection criteria, which is the use of passive sensing technologies, such as personal mobile and wearable devices, smart home sensors, and mobile applications, to unobtrusively monitor behaviors and physiological signals associated with cognitive functioning. Most studies (17, 56.7%^23–25,27–35,38–41,44^) focus on distinguishing between healthy individuals and those with MCI, with the majority of these works aimed at validating digital biomarkers or using sensor-derived metrics (e.g., sleep patterns, gait variability, keystroke dynamics) as predictors of cognitive decline. A complete detail of the results for this RQ can be found in Table S3 in the Supplementary Materials. Collectively, these studies demonstrate a strong emphasis on early detection and data-driven personalization, making meaningful contributions to the shift toward proactive, technology-assisted cognitive health assessment.

### Population RQ1: What are the characteristics of the population?

In order to answer PopRQ1, Table 1 summarizes the information about the population of each reviewed article (Table S4 in the Supplementary Materials includes detailed information). Clarifying the last six columns of Table 1, individuals with subjective cognitive impairment and no formal diagnosis of MCI or dementia were counted as CH, participants with reported mild MCI were counted as MCI cases, and lastly, cognitive impairment and AD diagnosis were included in the numbers of dementia cases.

**Table 1:** Size of the population used in each study reviewed, the ratio of female sex, age, and distribution of individuals between CH, low, and high AD risks, *amnestic MCI* (AMCI), MCI, and dementia

Reference of the reviewed study	Pop. *N*	Sex (% f.)	Age	% CH	% Low AD risk	% High AD risk	% AMCI	% MCI	% Dem.
Alam et al.^23^	17	88.23	M = 85.5, SD = 3.92	47	0	0	0	18	35
Lee et al.^24^	12	NR	M = 75.17, SD = 3.97	33	0	0	0	67	0
Chen et al.^25^	59	NR	NR	0	0	0	0	0	0
Xie et al.^26^	68	55.90	M = 67.48, SD = 4.89	44	0	0	56	0	0
Guarnieri et al.^27^	158	54.40	M = 70.47, SD = 12.79	56	0	0	18	0	27
Kimura et al.^48^	118	55.90	M = 75.7, SD = 5.8	0	0	0	0	100	0
Mc Ardle et al.^28^	108	40.74	M = 76.30, SD = 6.93	24	0	0	0	0	33
Lopez-Garcia et al.^52^	127	70.10	M = 65.47, SD = 6.33	100	0	0	0	0	0
Hossain et al.^51^	33	21.20	M = 54.18, SD = 4.02	67	0	0	0	18	15
Jung et al.^46^	108	62.00	M = 76.77, SD = 5.55	56	33	11	0	0	0
Eggenberger et al.^29^	80	62.50	M = 74.6, SD = 6.0	68	0	0	0	33	0
You et al.^30^	88	33.00	NR	40	0	0	0	60	0
Groznik et al.^31^	115	73.91	Mdn = 68, range = 43–94	46	0	0	28	17	10
Ghosh et al.^32^	33	48.00	M = 69.24, SD = 7.19	55	0	0	0	0	45
Liu et al.^33^	99	61.00	M = 66.69, SD = 8.16	31	0	0	0	69	0
Lee et al.^34^	42	69.00	M = 74.21, SD = 5.60	50	0	0	0	50	0
Lim^47^	18	72.00	NR	0	78	22	0	0	0
Alharbi et al.^35^	42	67.00	M = 72.95, SD = 5.87	50	0	0	0	50	0
Kimura et al.^49^	122	55.74	Mdn = 75.5, IQR = 71–80	0	0	0	0	100	0
Ghosal et al.^36^	86	50.00	M = 73.21, SD = 7.13	56	0	0	0	44	0
Ishibashi et al.^45^	117	100.00	NR	66	0	0	0	0	34
Yamada et al.^37^	54	50.00	M = 76.1, SD = 6.0	0	0	0	0	0	0
Park et al.^38^	595	52.60	M = 73.21, SD = 5.32	83	0	0	0	16	1
Ye et al.^39^	8116	43.00	Mdn = 63, range = 56–70	89	0	0	0	6	4
Eraslan et al.^40^	101	58.00	M = 70.72, SD = 6.91	33	0	0	37	0	31
Wang et al.^41^	122	47.50	M = 74.52, SD = 7.7	31	0	0	0	34	34
Winer et al.^50^	82,829	56.40	M = 62.0, SD = 7.8	100	0	0	0	0	0
Rykov et al.^42^	17	52.90	M = 60.3, SD = 4.5	0	0	0	0	100	0
Jo et al.^43^	76	64.50	M = 70.67, SD = 6.09	100	0	0	0	0	0
Bringas et al.^44^	35	NR	NR	0	0	0	0	20	80

NR stands for not reported and indicates that information was not found in the respective article. Articles are sorted according to the publication date (oldest at the first row). Some articles studied individuals with conditions not focused on this review (e.g., Parkinson’s disease), which are not represented, and may result in a total percentage lower than 100%.

Analyzing inclusion and exclusion criteria, we conclude that most of the reviewed research applied a minimum age of around 60 years and did not enforce any maximum age. Additionally, besides some study-specific diagnoses or test assessments, the second most commonly used criterion when selecting research participants was the ability to walk and perform *activities of daily living* (ADL) freely and independently. This may indicate a prevalence of approaches focused on the physical abilities of people throughout their lifespan. Looking into the exclusion criteria, most of those relate to health deficits or the occurrence of major health events like stroke in the past.

A direct result of those, and to summarize the sizes of the samples and the percentage of the female sex, Table 2 presents the mean, *standard deviation* (SD), median, minimum, and maximum values. It is important to note that three manuscripts did not report any gender or sex details. While some reported gender and other sex (at birth), we combined these into a single unique column. Overall, the sample sizes vary widely (ranging from 12 people to more than 82,000, with a median of 87), and the mean and median female ratios are similar, at around 56.5% of the cohort.

**Table 2:** Summary metrics of the size and sex distribution of the samples reported by the reviewed articles

	Mean	SD	Median	Minimum	Maximum
*N*	3120	15,126	87	12	82,829
Sex (% f.)	57.91	15.81	55.90	21.20	100

Three articles did not report any details on sex or gender.

Regarding the age of the populations included (see Fig. S6 in the Supplementary Materials), we can see that most of the articles reviewed focused on the ages between 65 and 80 years, with some SD bounds reaching 50 and 90 years. Regarding the disease or risk stages, the majority of the research included CH individuals and those with MCI.

Regarding the education years of the population used in the reviewed studies (see Fig. S7 in the Supplementary Materials), all the reported means are between 7.5 and 17.5 years, with some SD bounds reaching a minimum close to 2.5 years and a maximum of around 20. While the lower mean of 7.5 may represent samples mostly educated up to primary education, the higher mean of 17.5 indicates a possible completion of post-secondary education. These insights may refer to the diverse cultural and geographical origins of the reviewed studies, as revealed in the results of the previous subsection. However, they can also be linked to the varied age ranges across studies, which have been influenced by the inclusion criteria defined in each of them. Finally, only four (13.3%^29,31,43,49^) studies reported the APOE4 positivity of their participants, with most of them around 15% of the sample, indicating a low prevalence of genetic predisposition in the studied samples.

### Population RQ2: Are the datasets being used publicly available?

Almost none of the publications shared their data publicly (29, 96.7%, all except ref. ^50^), while 10 of those revealed the possibility of such sharing in case of a reasonable request (33.3%^26,29,32,33,38,40–42,51,52^). All research projects except two collected their data; the exceptions were the use of the “UK Biobank” by Winer et al.^50^ (publicly available) and the “CoSCo” dataset by Jo et al.^43^ (not shared). Overall, these results suggest that data sharing in this research domain remains far from being a standard. Table S5 in the Supplementary Materials provides a detailed description of the data-sharing policy per study.

### Methods RQ1: What is the approach taken by the researchers?

To better understand the most commonly used strategies for researching the screening and diagnosis of ADRD using DHTs, Table 3 details the modeling approaches, study designs, and the level of user interaction passivity with DHTs in the reviewed studies. All the reviewed manuscripts used data collected in participants’ or patients’ normal states without manipulation, which, in the context of this review, can be seen as an observational approach.

**Table 3:** Modeling approach, study design, and passivity of user interaction used in each of the reviewed research items

Ref.	Modeling approach				Study design		User interaction	
	Comparison	Correlation	Classification	Regression	Cross-sectional	Longitudinal	Entirely passive	Passive during active task
^23^		X			X			X
^24^			X			X	X	
^25^			X			X	X	
^26^		X				X	X	
^27^			X			X	X	
^48^		X				X	X	
^28^	X					X	X	
^52^		X				X	X	
^51^			X			X		X
^46^			X		X			X
^29^		X			X			X
^30^			X		X			X
^31^			X		X			X
^32^			X			X	X	
^33^			X			X	X	
^34^			X		X		X	
^47^			X			X	X	
^35^			X		X		X	
^49^			X			X	X	
^36^				X		X	X	
^45^			X		X		X	
^37^			X		X			X
^38^			X		X			X
^39^			X		X		X	
^40^	X				X			X
^41^			X		X			X
^50^		X				X	X	
^42^				X		X	X	
^43^	X				X		X	
^44^			X			X	X	

The modeling approach refers to how the DHT’s data and the outcomes were used, such as a direct comparison of outcomes between groups, statistical correlation analysis of outcomes with predictors, or the application of classification or regression techniques. Hereafter, we define “classification” and “regression” as they are defined in the literature on machine learning prediction. An example of the direct comparison approach is measuring differences in total sleep time between CH and AD patients. An example of the correlation approach is measuring the alignment between the amount of activity in a specific brain area and the advancement of cognitive decline components (e.g., memory). An example of the classification approach is predicting CH, MCI, or AD classes for a given patient, given DHT data. An example of the regression approach is predicting a continuous value for memory impairment with DHT data as input.

Most of the reviewed studies (19, 63.3%^24,25,27,30–35,37–39,41,44–47,49,51^) followed a classification methodology, focusing on using DHT’s data to classify individuals into different stages or conditions, as shown in Table 3. Correlation approaches were the second most frequent (six studies, 20.0%^23,26,29,48,50,52^), followed by direct comparison (three articles, 10.0%^28,40,43^), and regression (two articles, 6.7%^36,42^). However, even though regression approaches are almost as frequent as direct comparisons, we can see regression modeling being used more often in recent research, which may suggest an increasing interest in understanding the disease pathway of patients better, rather than simply categorizing them into predefined diagnosis categories.

Regarding the study design, 16 out of the 30 reviewed articles (53.3%^24–28,32,33,36,42,44,47–52^) followed their participants longitudinally, rather than only cross-sectionally. Given the timeline of search of this review being ten years (2014 to 2024), this prevalence of longitudinal research may be indicative of a recent (a) high access to consumer-grade DHTs, (b) high reliability of the DHTs applicable in this domain of investigation, (c) high acceptability of long-term usage of such DHTs by the overall population, or all of these.

Finally, this review focuses on DHTs that can or are already being used passively. For that reason, a third part of the answer to MetRQ1 must also contain an insight into the passiveness of the reviewed research. Overall, and in line with the prevalence of longitudinal methods, the majority (20, 66.7%^24–28,32–36,39,42–45,47–50,52^) applied an entirely passive data collection method. That means that any DHTs being used by those studies enabled continuous and user-interaction-free data acquisition. The remaining, as explained in the subsection “Eligibility of studies”, applied passive data collection during a specific task, which still required the active engagement of the participant for research purposes, but has the potential to evolve into an entirely passive application.

### Methods RQ2: What are the outcomes used as the gold standard, and how are they assessed?

As the studies reviewed focus on the use of DHTs for screening or diagnosing ADRD, following the decision on the modeling approach, researchers had to select the metrics against which DHTs would be compared, correlated, or used as the ground truth (reference measure) for classification and regression. Given the multitude of fields of the reviewed projects, those outcomes were grouped to match one of three possible main categories: identification of the stage of ADRD or healthy condition by technical or medical staff, metrics of cognitive decline (e.g., performance tests), and clinical ADRD biomarkers (e.g., medical imaging). Overall, most of the studies chose the disease stage diagnosis as the outcome to model or compare DHT against (17 articles, 56.7%^23–25,27–35,38–41,44^), followed by the usage of metrics of cognitive decline (nine articles, 30.0%^26,36,37,42,45–47,50,51^), and finally, the usage of clinical biomarkers of ADRD (four articles, 13.3%^43,48,49,52^). Table S6 in the Supplementary Materials presents detailed information on the outcomes used per study.

Among the studies that reported a formal diagnosis of AD as part of the reference standard, biological confirmation of amyloid pathology was inconsistently applied. One study^38^ relied on the 1984 NINCDS-ADRDA clinical criteria^54^, which did not incorporate biomarker evidence. Two studies^27,28^ referenced the 2011 NIA-AA criteria^55^, which incorporate amyloid biomarkers for a diagnosis of probable AD, although biomarker confirmation may not always be mandatory when evidence of neuronal injury is present. Two additional studies^40,44^ did not report the use of standardized diagnostic criteria and did not include amyloid or tau assessments in their diagnostic framework.

When analyzing the reported methods of assessing such outcomes, the primary category (stage of ADRD) used was primarily medical history, although some researchers indicated a study-specific clinical assessment. For the second (metrics of cognitive decline), Table 4 lists the areas focused on per research and helps us better understand which components of cognitive functioning are most studied by the nine articles using metrics of cognitive decline as an outcome. Memory is the most studied component, used by all nine projects, followed by attention levels, which are focused on by eight, and then language, used in five projects. Other less commonly used components include overall executive function (the manuscripts did not include subcomponent details), processing speed, registration, orientation, cognitive flexibility, planning, and visual perception. Some standardized instruments were often used to assess these outcomes: the *Mini-Mental State Examination* was employed by three studies, the *Digit Symbol Substitution Test* by one, the *ECog Scale* by another, and the *Trail Making Test* by another.

**Table 4:** Components and sub-components of cognitive functioning focused on by nine of the reviewed articles

Cognitive area evaluated (*N* of articles)	References
Memory (9, all)	^26,36,37,42,45–47,50,51^
Attention (8)	^26,36,37,45–47,50,51^
Language (5)	^26,37,46,47,51^
Executive function (4)	^26,36,42,50^
Processing speed (3)	^42,45,50^
Registration (3)	^46,47,51^
Orientation (2)	^37,51^
Cognitive flexibility (1)	^50^
Planning (1)	^37^
Visual perception (1)	^37^

Sorted from the most to the least used.

**Fig. 1:**
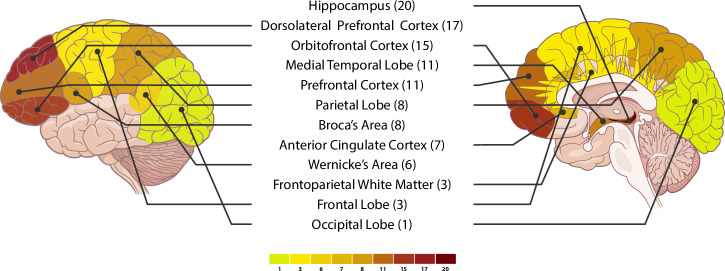
Brain regions focused on by the researchers when evaluating cognitive functioning (nine articles). The colors represent the number of times each region was explored. A single area may have been studied by more than one metric in a given study.

Mapping the brain regions targeted by the cognitive function assessment tools reported across the studies, Fig. 1 shows a heatmap of areas most frequently investigated. The hippocampus emerges as the most studied brain structure (20 times), consistent with the strong emphasis on memory as a cognitive domain. It is followed by the dorsolateral (17) and orbitofrontal (15) regions, both within the prefrontal cortex, reflecting the field’s focus on executive functions. Broader regions, such as the medial temporal lobe (11), parietal lobe (eight), and the general prefrontal cortex (11), are also commonly considered. The inclusion of language-related areas like Broca’s (eight) and Wernicke’s (six), as well as white matter structures (three), reveals attempts to capture a wide range of cognitive processes. This distribution aligns with established neurocognitive models of ADRD progression and highlights how DHTs are being designed to capture early and diverse patterns of cognitive decline.

### Methods RQ3: What types of DHTs are used to collect data, and what modalities are collected?

As the passive collection of data for screening and diagnosis of ADRD can be conducted in multiple ways, MetRQ3 aims to identify the most commonly used types of DHTs in this domain. For that, Table 5 organizes the reviewed manuscripts according to the overall category of the sensors used. Those categories included the commonly available accelerometer, photoplethysmogram (PPG) sensor for heart rate measurements, and temperature sensor found in consumer-grade smartwatches and fitness bands. Yet, the reviewed research also focused on less prevalent or available technologies such as bed sensors, body motion sensors, oximeters, shoe sensors, or even smart home devices. Overall, the accelerometer was the most used (17 articles, 56.7%^23,24,27,28,33,36,38,41,42,44,46–52^), followed by the PPG (9, 30.0%^23,24,33,35,42,43,48,49,51^), and by the temperature sensors (4, 13.3%^29,42,47,48^). These results may indicate a tendency towards daily-life devices, such as wearables or mobile-based ones, which also facilitate scalability. Table S7 in the Supplementary Materials presents further details on this information.

**Table 5:** Reviewed articles grouped according to the type(s) of sensor they employed

Type of sensor (*N* of articles)	References
Accelerometer (17)	^23,24,27,28,33,36,38,41,42,44,46–52^
PPG (9)	^23,24,33,35,42,43,48,49,51^
Temperature (4)	^29,42,47,48^
EDA (3)	^23,42,47^
Gyroscope (3)	^38,41,46^
Microphone (3)	^37,48,49^
EEG (2)	^34,39^
Eye tracker (2)	^31,40^
Altimeter (1)	^24^
Barometer (1)	^41^
Bed (1)	^25^
Blood Volume Pulse (1)	^42^
Body motion (1)	^30^
GPS tracker (1)	^32^
Magnetometer (1)	^41^
Oximeter (1)	^33^
Radar (1)	^45^
Shoe (1)	^26^
Smart home (1)	^23^

**Fig. 2:**
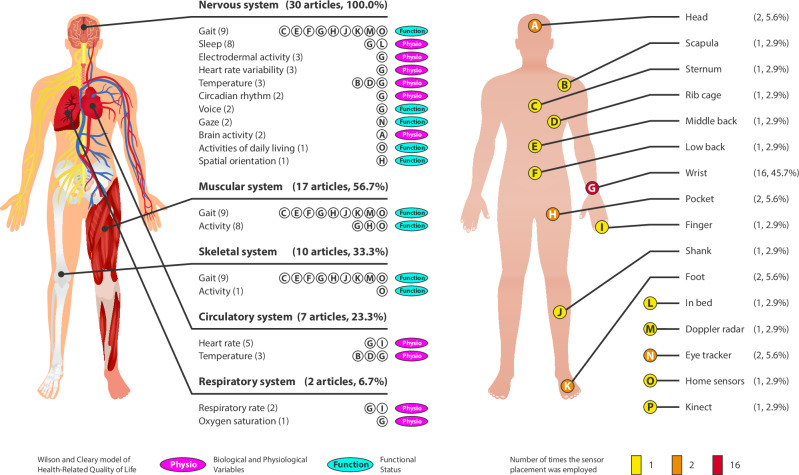
Human body systems focused on by the DHTs modalities used (left), its alignment with the Wilson and Cleary model of health-related quality of life (center), and placements of sensors on and around the human body (right).

Nevertheless, certain devices within the same category could have been employed by researchers to collect data on various modalities (e.g., an accelerometer could measure activity, gait, or sleep patterns). To facilitate a comprehensive understanding of the reviewed research, Fig. 2 illustrates the distribution of these devices across the distinct systems of the human body. Additionally, it aligns them with the corresponding group within the Wilson and Cleary model of health-related quality of life outcomes (referenced in ref. ^56^). Furthermore, the figure depicts the body region on or around which the sensors were utilized.

Unsurprisingly, all 30 articles focused on modalities of the nervous system, followed by the muscular system, indicating a strong focus on the brain’s abilities over time and the patients’ physical capabilities. Examining the specific data modalities, gait was the most assessed (nine articles, 30.0%^26,28,30,36,38,41,44–46^), followed by physical activity and body temperature. Regarding the health-related components of quality of life, biological and physiological variables (i.e., how the body functions automatically or unconsciously) were slightly more explored (10 out of 19 cases, 52.6%), while functional status (i.e., how a person consciously performs activities) was more predominantly used (e.g., gait was the most used modality, increasing the significance of this model component). Finally, when analyzing the body location where the DHTs were placed, wristworn devices stood out clearly (16 times, 45.7%^23,24,27,28,33,36,37,41–43,47–52^), which may indicate a strong focus on research using consumer-grade technologies that are easy to wear in daily life. Devices placed on the head, pocket, foot, or around the patient using eye tracking were all the second most used body placement, potentially revealing a lower but important usefulness of mobile technologies (placed in the pocket) and of single-focus approaches (brain activity, gait, and gaze).

### Methods RQ4: What are the characteristics of data collection?

To better identify the trends in the data collection settings used, the MetRQ4 focuses on duration, frequency, periods of data sampling, data validity minimums, and other aspects reported by the researchers.

Analyzing the data collection periods, places, and sampling frequencies, we find that the number of studies with data collection anywhere (13, 43.3%^24,26–28,32,33,36,42,47–51^) and in laboratory settings (12, 40.0%^29–31,34,35,37–41,45,46^) is almost equal, with the ubiquitous approaches predominating. Following a similar trend, the most used frequency of data gathering was continuous (15, 50.0%^24–28,32,33,35,36,44,45,47,49–51^), with the second most frequent being “once” (12, 40.0%^23,29–31,34,37–41,43,46^). Additionally, studies started data collection as early as 2009, while most of them focused on periods after 2016. Figure S8 in the Supplementary Materials presents this information in a summarized way, and details can be found in Table S8 in the Supplementary Materials.

Additionally, by looking at Fig. S9 in the Supplementary Materials, the most frequent duration of data collection was one week (7^27,28,36,44,47,49,50^, 29.2% of the research that reported such information, 24 articles), followed by two weeks (3, 12.5%^26,32,33^) and by one night (2, 8.3%^39,43^). However, it ranged from 2 minutes to 6 months, with seven options found to be under a day. Regarding follow-up duration (how long a participant was followed with interrupted data acquisition), only three studies (10.0%^48–50^) applied one, which ranged between one and three years. Table S9 in the Supplementary Materials contains detailed information on this matter.

Due to the passive nature of the data used in the reviewed research, missing data and low-quality information may have been collected. To prevent interference with research results, data validity thresholds may have been implemented, which refer to the minimum amount or quality of data required for it to be used in the analysis. Overall, only seven of the 30 manuscripts (23.3%^25,26,28,33,34,42,50^) reported the application of such, and only three (10.0%^39,42,50^) informed on the data imputation applied. When applying data validation, researchers mostly focused on minimums around 50% of the data collection total duration, with ranges between around 40% and 70%. Regarding data imputation techniques, although the number of articles reporting having used them was too low, the ones that used them applied nearest neighbor imputation or linear interpolation methods. A comprehensive detail on the data validity thresholds and data imputation applied by researchers can be found in Table S10 in the Supplementary Materials.

### Methods RQ5: Which techniques and machine learning algorithms are used in analyzing data from DHTs for the screening or diagnosis of ADRD?

Comparison approaches (three articles) mainly focused on the analysis of variance (ANOVA) and evaluated the statistical quality (i.e., level of evidence) of their results using *p*-values. Regarding correlation research (six articles), feature selection was only applied in one article (functional PCA^50^). The method used most frequently was Pearson correlation (two articles), but regression options were also explored. Confounders included mainly age and sex aspects, and the evaluation was more often performed using *p*-values and correlation coefficients. Table S11 in the Supplementary Materials provides a detailed description of the feature selection methods, controls, and evaluation metrics employed.

For classification (19 articles), feature selection was primarily applied using random forest-derived methods, and modeling was mainly performed with logistic regression or support vector machines (although some deep learning was also employed in some instances), while adjusting for confounders similar to those used in correlation procedures. Cross-validation in these cases was primarily done in 10- or 5-fold. Regarding machine learning regression approaches (two studies), only one study performed feature selection, using Pearson and Spearman correlations^42^. The modeling method was either linear or employing elastic net, random forest, or extreme gradient boosting, the cross-validation was 10-fold or leave-one-out, and the confounders used in one study were consistent with those in other cases (age and sex). Table S12 in the Supplementary Materials contains detailed information on these two approaches.

As for the evaluation metrics used in the classification approaches, the most used was sensitivity (also referred to as recall, 15 articles, 78.9%^25,27,30,32–35,37,41,44–47,49,51^), followed by accuracy (13, 68.4%^25,27,30,31,33–35,37,38,44,45,47,51^) and precision (9, 47.4%^25,27,33,44–47,49,51^). On average, this type of research used 3.3 metrics combined per article, with a mode value of three (used by six studies), followed by four (five cases) and two (four cases). For regression, the two studies reported either R-squared or *mean absolute error* (MAE) values. Table S13 in the Supplementary Materials contains detailed information on this.

### Evaluation RQ1: How accurate and reliable are passive DHTs in screening or diagnosing ADRD in non-clinical settings?

As the reviewed research followed four distinct approaches, we hereby answer EvaRQ1 for each of them: comparison, correlation, classification, and regression. For the comparison approaches, the three studies (10.0%^28,40,43^) found statistically discernible (i.e., statistically significant^57–60^) differences between CH and AMCI and dementia patients when leveraging gait, sleep, and eye tracking data modalities. The between-group differences were only studied in relation to gait and sleep data, and included a minimum of 0.083 for walking time and bouts per day, and a maximum of 0.138 when comparing the mean bout length between CH and PwD, as well as a 10.1 min difference in total time of sleep in the slow-wave phase. Table S14 in the Supplementary Materials presents these results in detail.

Regarding research articles that employed correlation analysis, researchers in all six articles (20.0%^23,26,29,48,50,52^) identified statistically significant links in the data that could be used to distinguish between CH, aMCI, MCI, dementia diagnosis, and their underlying pathology. The data used encompassed various aspects, including gait, eye tracking, ADL, *electrodermal activity* (EDA), heart rate, and sleep. In relation to physical activity, walking velocity, stride length, and stride time variability were found to be significantly associated with long-term memory, executive function, and attention. Conversely, conversation time was positively correlated with cerebral glucose metabolism, while *wakefulness after sleep onset* (WASO), total sleep time, and walking time were negatively associated. Additionally, total sleep time was negatively correlated with total tau protein deposition. Furthermore, daily heart rate metrics and cognitive function were linked at specific moments of the day. Skin temperature of specific body regions was also correlated with cognitive functioning. Lastly, EDA and ADL revealed a link with CH and MCI states. A comprehensive list of results is provided in Table S15 in the Supplementary Materials.

**Fig. 3:**
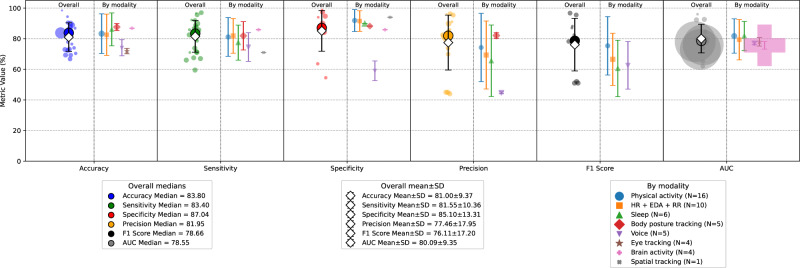
Distribution and subgrouped performance of classification metrics across included studies. Accuracy, sensitivity, specificity, precision, F1 score, and AUC are shown for the 19 articles that performed classification. For each metric, the left section (“Overall”) displays individual study results with median and mean ± SD summaries, while the right section (“By modality”) presents mean ± SD values aggregated by digital health technology modality. All reported metrics are derived from in-sample cross-validation and do not necessarily reflect out-of-sample generalizability. Symbol sizes are proportional to the average population size contributing to each estimate.

Finally, considering the articles reviewed that focused on classification^24,25,27,30–35,37–39,41,44–47,49,51^ or regression methods^36,42^, Fig. 3 shows the distribution, means, SDs, and medians of each of the six most-used metrics of evaluation in the classification case. When multiple models were being used and their results being reported in an article, we represent each of them with a different group of dots in the chart. Tables S16 and S17 in the Supplementary Materials supplement this information for both cases.

DHTs enabled a classification approach with median accuracy, sensitivity, specificity, and precision ranging from 80% to 90%, while the F1 score and AUC had a median of approximately 78.5%. Precision and F1 score had the largest SDs of the six metrics. It is important to note that all reported performance values reflect in-sample cross-validation rather than external validation on independent cohorts. As such, these metrics represent internal consistency and should not be interpreted as estimates of real-world generalizability.

Additionally, analyzing the performance of the regression approaches (two), DHTs are also useful to predict several components of cognitive function. Attention, verbal memory, and executive function were predicted with an R-squared of 0.214, 0.383, and 0.414, respectively, in a case of possible limitation of the modeling technique selected (linear regression). Finally, global cognition could be predicted with an MAE as low as 0.09.

Overall, answering the EvaRQ1, we can observe that leveraging DHTs data leads to a high accuracy in screening or diagnosing ADRD in non-clinical settings. Such results depend on the approach selected; however, considering classification evaluation metrics, the accuracy, sensitivity, specificity, and precision are high enough to conclude that DHTs are on the path of becoming a reliable method for achieving this task, despite external validation being lacking at the moment in most of the research found.

### Evaluation RQ2: What specific components/sources of the used DHTs are most informative of ADRD?

To better understand which sources of DHTs are most predictive when screening or diagnosing for ADRD, we first organized each of the 19 articles that performed classification into groups based on the type of DHTs’ modalities used as input for the methods. Regression approaches could not be mapped together because they did not report similar evaluation metrics. These groups were “Physical activity” to represent behavior function, “HR + EDA + RR” to represent biological and physiological variables (heart rate, EDA, and respiratory rate), “sleep,” “body posture tracking” to represent devices capable of wirelessly track one’s posture when moving, “eye tracking,” “spatial tracking” for cases of GPS-powered appliances, “brain activity” for methods using EEG or similar technologies, and finally the group “voice.” Figure 3 presents the mean and SD of each group’s performance over the six most used evaluation metrics, with the number of data points for each group indicated in the legend to the right (multiple groups could be focused on at the same time). Because the size of the dataset used could have influenced the results, the symbol size for each group is proportional to the mean population sample size in the studies reviewed in that group.

Physical activity modalities led to the highest mean F1 score and AUC. Body posture tracking enabled the highest mean accuracy and precision. Spatial tracking and brain activity modalities resulted in the highest mean specificity and sensitivity, respectively. However, considering the upper bound of the SD of each group, physical activity outperforms all other groups in all six metrics, closely followed by the “HR + EDA + RR” and sleep modalities.

### Implementation RQ1: How do individuals perceive and interact with DHTs, and what design features and user interfaces relate to adherence and acceptance?

Understanding to what extent the individuals (patients or not) perceived and interacted with the DHTs used in each of the reviewed articles, we found only one of the 30 manuscripts addressing this in its text. Liu et al.^33^ reported that some users stopped wearing the smartwatch due to discomfort, housework, and other reasons.

### Implementation RQ2: How can data privacy and security be ensured while enabling effective screening and diagnosis?

A truly critical question to be answered when dealing with continuous, passive, and ubiquitous monitoring of one’s life is addressed in only one of the 30 articles. Interestingly, that same manuscript was the only one to employ GPS tracking of a device with the patient at all times^32^, which involves highly sensitive and private data.

### Implementation RQ3: How do lifestyle factors and environmental conditions affect the data collected by DHTs?

Regarding factors that authors revealed having interfered with the data collection, only four (13.3%^25,33,35,42^) of the reviewed manuscripts included details on this aspect (not to be confused with the controls used and described above). Chen et al.^25^ noted that day napping data would reduce the model’s performance and so proceeded with removing them from the analysis. Liu et al.^33^ noticed that daily routines involving, for example, cleaning the house spaces, could interfere with the wearable data collected from time to time. Alharbi et al.^35^ reported that some HRV measurements could have been affected by changes in circadian rhythm, hormonal shifts, and acute stressors throughout the day. At last, Rykov et al.^42^ also addressed the limitations of wearable devices, such as the loss of contact between sensors and the skin surface.

Taken together, these observations suggest that DHT data is highly context-sensitive, and that failing to account for environmental and lifestyle variability may undermine the reliability and validity of downstream analyses. As such, future work should prioritize the systematic identification and modeling of such influencing factors, either through improved preprocessing strategies, additional context-aware sensors, or adaptive algorithms that can account for behavioral and physiological confounders in real-world, unsupervised settings.

### Implementation RQ4: Does the use of DHTs in non-clinical settings impact the timing and accuracy of ADRD screening and diagnosis?

To answer ImpRQ4, we first extracted the sentences (or parts of) from the reviewed manuscripts where authors referred to the impact of using DHTs on the timing and accuracy of ADRD screening and diagnosis. This resulted in 78 sentences extracted from 27 of the 30 reviewed articles (90.0%, all except^24,40,45^, with a mean of 2.9 sentences per article), which we concatenated (totaling 1,047 words, with a mean of 38.8 per article). The resulting ten most frequently used words are presented in Table 6. The ten most frequent terms refer to the research goal (“monitoring,” “cognitive,” “risk,” “early”), to medical conditions (“MCI,” “AD”), and to the DHT modalities and formats (“gait,” “wearable,” “sleep”). Table S18 in the Supplementary Materials contains all the extracted sentences per article.

**Table 6:** The most frequent ten words in author expressions linked to the impact of DHTs usage on the timing and accuracy of ADRD screening and diagnosis

Word	Times used	Percentage (total = 1047)
Monitoring	19	1.81%
Cognitive	18	1.72%
MCI	14	1.34%
Gait	11	1.05%
Wearable	11	1.05%
Risk	11	1.05%
Sleep	10	0.96%
Early	10	0.96%
AD	10	0.9%
May	10	0.96%

The prominence of words such as “early,” “monitoring,” and “risk” suggests that DHTs are primarily viewed as tools that enhance the timeliness of ADRD screening by enabling earlier identification of subtle behavioral and physiological changes indicative of preclinical or prodromal stages. Meanwhile, the focus on specific modalities, such as gait and sleep, particularly through wearable devices, highlights a shift toward continuous, ecologically valid data collection, which supports the impact resulting from greater diagnostic precision. Taken together, these findings suggest that researchers view DHTs not merely as supplementary tools but as enablers of earlier and perhaps more accurate detection of cognitive decline, potentially leading to more timely interventions and improved clinical decision-making.

### Synthesis of sensing modalities, cognitive targets, and temporal trends

Beyond the descriptive results presented in the previous subsections, we conducted an integrative synthesis to identify broader structural patterns in how passive DHTs have been deployed across cognitive domains, sensor modalities, and temporal research trends. This synthesis combines information from *research questions* (RQ) MetRQ2-3 and MInfRQ1 to highlight convergent themes that are not apparent when examining each dimension in isolation.

Summarizing how different sensing modalities align with the cognitive domains assessed in the nine reviewed studies that used metrics of cognitive decline as a gold standard (see Fig. S10 in the Supplementary Materials), a clear pattern emerges: activity, gait, sleep, and heart-rate-derived signals dominate memory, language, and attention assessments—domains characteristically affected early in ADRD. More specialized modalities (e.g., voice, EDA, temperature) appear in fewer cognitive contexts, reflecting their early exploratory status.

**Fig. 4:**
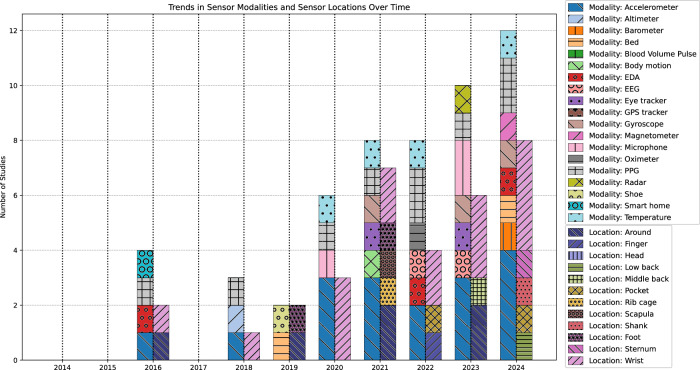
Temporal evolution of sensor modalities and sensor locations across included studies. For each year, the left stacked bars represent the number of studies using each sensor modality, while the right stacked bars represent the corresponding sensor placement locations. Each segment reflects the frequency of occurrence of a given modality or location within that year across the 30 reviewed studies.

Figure 4 illustrate how sensor technology and sensor placement evolved over the years. While accelerometers and PPG remain the most frequently used modalities, later years show a diversification toward EEG, radar, and multi-sensor systems. A parallel trend appears in sensor placement: although wrist-worn devices were consistently dominant, recent years demonstrate increased experimentation with foot, shank, trunk, and pocket placements, aligning with growing interest in multimodal mobility and physiological signatures.

Together, these integrated analyses reveal a field transitioning from narrowly focused, single-modality pilots toward richer, multimodal sensing ecosystems aimed at capturing subtle behavioral and physiological markers of early cognitive decline.

## Discussion

Although we adhered to PRISMA guidelines and included data from six major databases, it is important to acknowledge that our synthesis may have certain limitations that readers should be aware of. First, we restricted the search to English-language papers published between 2014 and July 2024. Consequently, relevant work in other languages or earlier proof-of-concept studies may be absent, potentially skewing the perception of a recent publication’s rapid impact. Second, we relied on title-and-abstract screening followed by an introduction-and-conclusion scan for full-text exclusion. Although there were always two reviewers who cross-checked decisions, we did not conduct a formal third-party adjudication, which might have resulted in some borderline studies being discarded in error. Third, the heterogeneity of study designs, sensor types, and outcome definitions prevented a quantitative meta-analysis; instead, we summarized performance descriptively, which cannot account for sampling variance. Fourth, we extracted accuracy metrics as reported: many papers used different class balances, cross-validation folds, or test splits, making across-study comparisons approximate at best. Fifth, our decision to include studies that actively collected data but simultaneously advocated for future passive deployment may have expanded the scope while simultaneously introducing speculative elements. Finally, all data extraction was limited to information present in the published manuscripts, leaving potential gaps in unpublished protocols, code, or supplementary results.

Reflecting on the fact that passive DHT has gained significant traction in the past five years, particularly in the context of ADRD screening and diagnosis, the majority of the 30 studies reviewed here were published after 2019, with a substantial number appearing in top-quartile computer science or medical journals. This trend suggests a transition from a niche area of research to a mainstream scientific discipline. Advancements in sensor technology, extended battery life, and increased funding dedicated to early ADRD detection have likely contributed to this progress.

During the covered period, the majority of the data originates from teams located in Europe and East Asia, regions characterized by rapidly aging populations and robust technological infrastructure. This concentration of data is understandable for proof-of-concept endeavors. However, it also implies that the current algorithms may have been trained on a relatively limited spectrum of humanity. As consumer-grade DHTs (such as wearable devices) become more affordable and telecommunication coverage expands, we anticipate an increase in studies conducted in North America (which, for the moment, is not as represented as it would be expected given its technological innovation), in Africa, Latin America, and other underrepresented regions. These studies should encompass a more diverse range of demographics and lifestyles, thereby contributing to a more comprehensive understanding of the subject matter and the specific life choices that influence the incidence of ADRD.

The datasets used in the reviewed research items exhibited a certain degree of heterogeneity. The population sample sizes ranged from a minimum of 12 to a maximum of 82,000, with a median of 87. Participants’ average age was approximately 70 years, and on average, more than half were female. With respect to disease stages, most of the studies involved CH and MCI patients, followed by dementia cases. Notably, only two projects utilized public repositories, potentially indicating the innovative data collection methodologies necessitated in this field. In terms of data sharing policies, only ten projects demonstrated availability for sharing upon request, while the remaining retained their data private, thereby hindering transparency, replication, and collaborative efforts on joint datasets. To facilitate open science collaborations, data-sharing mandates from funding entities and journals should encourage the community to adopt open, FAIR-compliant datasets and public leaderboards.

Methodologically, the majority of published studies adhere to a well-established approach: passively collect wrist accelerometer and photoplethysmography data streams, select features using a random-forest technique, and subsequently input these features into logistic regression or support vector machine models that undergo validation through five- or ten-fold cross-validation. While approximately half of the monitored studies conducted long-term assessments, spanning several weeks or months and continuously gathering data, only seven explicitly specified a data-quality threshold, and only three provided detailed explanations for handling missing data. As the size of these cohorts increases, our findings, in conjunction with prevailing research trends in computer science, suggest a potential shift toward self-supervised and deep learning-based models that directly learn from raw time series data. Furthermore, the primary focus of the models was on classifying patients’ data into disease stages (CH to dementia). Only two of the 30 research items employed regression modeling capable of predicting a patient’s progression along the cognitive decline process. These publications, which occurred in 2023 and 2024, suggest a potential shift toward a more continuous estimation of brain health components rather than simplistic diagnostic categories. This shift potentially acknowledges the necessity of personalized and dynamic follow-up and therapeutic interventions. Subsequently, the utilization of metrics for cognitive decline was undertaken, with memory, attention, and language being the most extensively studied. This emphasis on memory and executive functions is mirrored in the neuroanatomical focus of the studies, with the hippocampus, prefrontal regions, and temporal lobes being the most frequently targeted areas, highlighting the alignment between cognitive theory and the neurobiological substrates monitored by DHTs. This convergence suggests that DHTs may be particularly suited to detect subtle changes in memory and language—domains most affected in the early, often asymptomatic, phases of cognitive decline.

The cross-domain synthesis reinforces this pattern, showing a strong alignment between the cognitive domains most affected in early ADRD and the sensing modalities most commonly deployed. Activity- and gait-derived signals were consistently mapped to memory, language, and attentional assessments, domains that deteriorate earliest in prodromal stages. This convergence suggests that, even without explicit coordination, the field has organically gravitated toward passive sensing modalities that capture the behavioral and physiological pathways most tightly coupled to early cognitive decline. At the same time, modalities such as voice, EDA, and temperature appeared in a smaller number of cognitive contexts, reflecting their current exploratory status and pointing to potential opportunities for broader cognitive coverage in future multimodal sensing frameworks.

Despite the limited sample sizes in the population across studies, the overall model performance was robust. Median accuracy, sensitivity, specificity, and precision consistently exceeded 80%, while median F1 score and AUC were slightly below this threshold. Among the various DHT modalities, physical activity data exhibited the highest mean F1 score and AUC. Body posture tracking achieved the highest mean accuracy and precision, while spatial tracking and brain activity demonstrated the highest mean specificity and sensitivity, respectively. However, when considering the upper bound of the SD across modalities, physical activity outperformed all other modalities in all metrics. Sleep and physiological signals (heart rate, EDA, respiratory rate) also exhibited competitive and consistent performance, closely approximating physical activity. While these accuracy levels are encouraging, they largely stem from in-sample, cross-validated analyses rather than true external validation. This distinction is critical: cross-validation assesses internal reproducibility but does not guarantee that the same models would perform similarly on independent cohorts or in different real-world contexts. The almost complete absence of external validation across the reviewed literature, therefore, limits the extent to which performance claims can be generalized.

Aligned with robust models’ performance, temporal trends in modality adoption (Fig. 4) further illustrate the field’s maturation. Early studies relied almost exclusively on accelerometry and heart-rate photoplethysmography, reflecting the capabilities of first-generation consumer wearables. After 2020, however, the introduction of radar, EEG, magnetometers, respiratory sensors, and more sophisticated multimodal devices demonstrates a decisive shift toward richer physiological characterizations. This diversification indicates that the field is transitioning away from isolated behavioral proxies toward integrated sensing ecosystems capable of capturing multiple dimensions of cognitive and functional decline.

In contrast, the practical application of this research field remains a challenge, primarily due to human factors that influence data gathering. Despite their importance for real-world deployment, user experience and privacy remain largely unexplored in the current evidence base: only one study in our review explicitly evaluated usability or user perceptions^33^, and only one examined privacy considerations^32^. This gap likely reflects the field’s early emphasis on demonstrating predictive feasibility and algorithmic performance, as well as the absence of standardized frameworks for evaluating acceptability, burden, and data-governance implications in passive sensing for ADRD. In addition, reporting of potential confounding variables, such as comorbidities, medication use, socioeconomic characteristics, environmental exposures, and device adherence, was inconsistent across studies. Attrition patterns and missing data mechanisms were rarely described in sufficient detail to permit structured cross-study evaluation. Consequently, systematic analysis of confounding effects or selection bias was not feasible within the constraints of the available evidence, representing a broader reporting gap in the field rather than a limitation of the present review. The evolution of sensor placement shown in Fig. 4 also reflects growing attention to ecological validity and signal specificity. While wrist-worn devices remain dominant due to comfort and widespread adoption, newer studies increasingly incorporate foot-, shank-, torso-, or pocket-mounted sensors to capture gait, mobility, postural control, and cardiopulmonary dynamics—physiological processes known to degrade subtly in preclinical ADRD. This methodological expansion suggests a shift toward more granular and context-aware sensing that may improve early signal detection while capturing functional aspects of daily life that wrist sensors alone cannot fully characterize. Future research would benefit from incorporating established methodologies from human-centered design and usability research, such as iterative co-design processes, structured usability assessments, and long-term adherence evaluations, as well as privacy and security frameworks such as GDPR-aligned data minimization and purpose-limitation principles. Integrating these dimensions will be crucial for ensuring that passive sensing technologies are not only accurate but also acceptable, trustworthy, and sustainable in real-world care pathways.

Beyond user experience and privacy, equity must also be treated as a core determinant of real-world feasibility. Although several studies acknowledged region-specific recruitment, none examined whether passive sensing technologies function equitably across diverse socioeconomic, cultural, or digital-access contexts. This lack of reporting reflects a broader structural gap: as long as evidence is generated primarily from highly resourced, technologically connected populations, it remains unclear whether the observed performance generalizes to individuals with different mobility patterns, housing environments, health literacy levels, or technology access. Equity-sensitive deployment of passive sensing will therefore require frameworks that explicitly address accessibility, burden, digital literacy, affordability, and cultural acceptability. Future studies could draw on principles of digital health equity, inclusive-by-design methodologies, and implementation-science approaches to ensure that early detection systems do not inadvertently worsen existing disparities in dementia diagnosis or care. In the future, it is highly probable that regulators and patients will eventually demand more comfortable or even textile sensors, on-device processing, differential privacy safeguards, and study designs that comprehensively assess adherence and trust from the outset.

Taken together, the evidence compiled in this review shows a field that is rapidly evolving from technologically constrained, modality-specific pilots toward coherent sensing architectures aligned with ADRD pathophysiology, daily functioning, and real-world feasibility. The increasing diversity of modalities, body placements, and cognitive targets reflects not fragmentation but convergence toward a multidimensional representation of early cognitive decline; one that may ultimately support more sensitive, personalized, and continuous risk assessment. In this trajectory, privacy-preserving sensing networks could feasibly detect subtle abnormalities months before conventional clinical encounters, enabling earlier referral to memory clinics and enriching longitudinal data for therapeutic trials. Realizing this potential will require transparent methodologies, harmonized reporting standards, and robust evaluation frameworks to ensure that emerging passive DHT systems can be deployed reliably, ethically, and at scale.

To help the field move from promising prototypes to reliable, equitable, and clinically actionable tools, we propose a clear set of priorities. First, transparency and harmonization must be strengthened, including standardized reporting templates, consistent study designs, explicit data-quality criteria, and unified outcome definitions. As part of this foundational step, we highlight the need to identify and consistently measure core data streams that the literature most strongly supports, namely accelerometry-derived activity and sleep-wake patterns, and photoplethysmography-derived heart rate and heart-rate variability, given their demonstrated predictive value and their evolution from earlier reliance on Patient-Reported and Performance-Reported outcomes assessments toward more temporally dense passive measures. Second, broader population coverage and equity should be actively pursued by expanding recruitment to underrepresented geographical regions, socioeconomic contexts, and demographic groups. Third, once these foundations are in place, larger datasets, multimodal sensing, and more advanced yet interpretable and privacy-preserving modeling techniques will be best positioned to generate robust and generalizable evidence. Finally, usability, adherence, security practices, and user perceptions must be evaluated alongside algorithmic performance to ensure real-world feasibility. Taken together, these steps can transform passive DHTs into a low-burden, scalable front-end for early ADRD triage and clinical-trial enrichment; complementary to, though not yet a replacement for, comprehensive clinical evaluation.

## Methods

We followed the recommendations made by Silva et al.^61^ when performing this systematic literature review, according to the *Preferred Reporting Items for Systematic Reviews and Meta-analysis* (PRISMA) statement^62,63^.

### Search strategy

This review was performed using six libraries: IEEE Xplore, ACM Digital Library, PubMed, PsyNet, Scopus, and Web of Science. The first two were chosen as they include most of the high-quality computer science research results. PubMed and PsyNet were used to include publications in the medical and psychological fields, respectively. Finally, the last two libraries were adopted due to their relevance in multi-field research. This search covered research published and indexed between 1 January 2014 and 9 July 2024, spanning roughly a decade and a half of work in this field. This ten-year window aligns with common practice in technology-focused systematic reviews and corresponds to the period during which consumer-grade wearables (e.g., accelerometer- and photoplethysmography-enabled devices) and smartphones with dedicated health sensors achieved widespread adoption^64^. This technological turning point, occurring approximately between 2014 and 2016, substantially expanded the feasibility of continuous passive data collection in real-world, non-clinical environments. It was performed using the search string presented below, which matched the titles and abstracts of available articles.

(wearable* OR “ubiquitous technology” OR smartwatch* OR tracker*)

AND

(Alzheimer* OR “AD” OR “cognitive impairment” OR “MCI” OR “dementia”)

AND

(screen* OR diagnos* OR “early detection” OR monitor* OR forecast*)

The search terms were developed based on our domain expertise and prior review experience in DHTs, aiming to strike a balance between breadth and relevance. We designed a concise Boolean query that could be applied consistently across all selected databases. This approach was necessary because some database platforms do not support long or highly nested Boolean expressions. Using a unified and streamlined query ensured methodological consistency across heterogeneous sources while still capturing the breadth of work on passive sensing for ADRD.

### Study selection and quality evaluation

The search results returned a total of 1608 articles, of which 551 were duplicates and were removed. The focus of our work aimed to include only articles following the inclusion and exclusion criteria in Table 7. According to those, we only included articles written in English, reporting on interventional or observational research on humans 18 years or older, CH or with ADRD identified/diagnosed, published after peer-review of journals or conference proceedings, applying wearable or ambient sensors’ passive data collection for a usage in a non-clinical environment, and aiming at the screening, diagnosis, or similar, of ADRD.

**Table 7:** Inclusion and exclusion criteria used in this review

Type	Inclusion	Exclusion
Publication date	Between 1 January 2014 and 9 July 2024	Before 1 January 2014
Geographic location of the study	All	None
Language	English	Any other
Population studied	Humans	Any other
Population age	18 or older	Younger than 18
Population’s cognitive state	CH or with diagnosed ADRD	Diagnosis of any impairment, non-AD-related
Study design	Interventional or observational	Any other
Type of publication	Peer-reviewed journal or conference	Any other (including preprint, thesis, abstract, poster)
DHT usage in the real world	Wearable or ambient sensors with passive data collection	Any other (e.g., only lab- or clinic-based)
Study context or applicability of results	Non-clinical environment	Only the clinical environment
Research goal	Screening, diagnosis, or similar of ADRD	Monitoring or other

A screening phase then followed, divided into three rounds, during which we made use of the *Rayyan QCRI*^65^ online platform as support for collaboration. During the first round, three authors (I.M., P.P., and E.J.D.) analyzed the titles and abstracts of the eligible articles and included, excluded, or doubted them. The first author examined all the articles, while the second and third authors analyzed half of them, meaning that two authors considered each eligible manuscript. The ones with included and doubted inclusion were further examined by all three authors again in the second round. That second round involved examining both the introduction and conclusion of the manuscripts, following the same split of articles among the same three authors. In case of disagreement on the decision for a specific article, a screening of the paper was made until a common inclusion/exclusion agreement was reached. By the end of the two rounds, 89 manuscripts were included in the third and final step. From those, the full manuscript of two of them could not be obtained (the corresponding authors were contacted, but there was no reply within 15 days).

A full read of the introduction and conclusion, by the first author, then led to the further exclusion of 57 articles. Eighteen articles were excluded because they did not include a cognitive screening or diagnostic outcome, instead focusing on related but distinct aims such as gait subtype characterization, wandering detection, or mobility prediction. Seventeen studies relied exclusively on active data collection without passive sensing. Nine records did not present extractable results, and six focused solely on developing post-diagnosis monitoring tools rather than screening or early detection. An additional five articles were excluded due to insufficient methodological reporting to enable data extraction, including missing population descriptions, unclear inclusion criteria, or absent outcome definitions or performance metrics. Finally, two records were excluded due to an ineligible publication type (e.g., preprint).

**Fig. 5:**
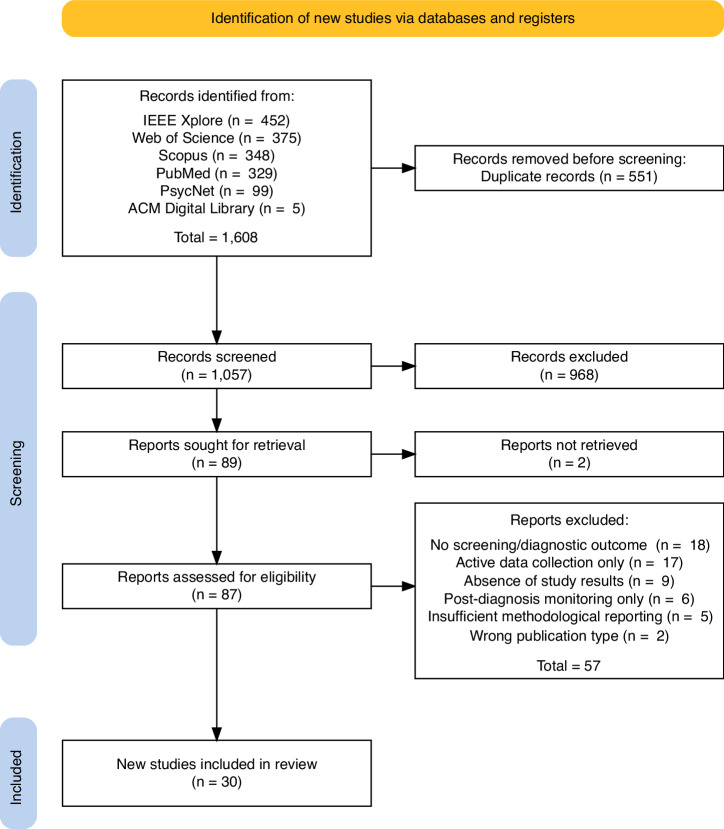
PRISMA flow diagram of identification and inclusion of papers in this review.

Finally, 30 articles were included in this review, as the diagram in Fig. 5 depicts. For clarity, all included studies reported extractable results directly relevant to cognitive screening or diagnosis. None were included solely on the basis of speculative or future applications; any references to future work in the manuscript refer only to potential real-world deployment, not eligibility.

We assessed methodological quality using the standard NOS, which assigns up to nine stars across the Selection, Comparability, and Outcome domains. Each study was first classified, by I.M. and K.W., as a cohort or case-control design to apply the appropriate NOS rubric. The same authors then independently evaluated all studies in a blinded manner, scoring each NOS domain separately. Discrepancies were resolved through discussion to arrive at a final consensus rating for each study. NOS scoring focused exclusively on the quality of participant recruitment and diagnostic ascertainment procedures, independent of the digital technology tested.

### RQ

We followed Kitchenham’s^66^ suggestion when defining this review’s RQ. Thus, we established our RQs based on their five perspectives, inspired by the work of Siebra et al.^67^.

Metainformation questions are based on the metadata of the reviewed articles:

- MInfRQ1: When, where, and by whom was the article published?
- MInfRQ2: What is the objective and scientific contribution?

Population questions focus on the details of the population studied and data sharing policies:

- PopRQ1: What are the characteristics of the population (e.g., size, demographics)?
- PopRQ2: Are the datasets being used publicly available?

Methods questions refer to the technical features and modeling approach employed by the work reviewed:

- MetRQ1: What is the approach taken by the researchers (e.g., longitudinal or cross-sectional, observational or interventional, classification or regression)?
- MetRQ2: What are the outcomes used as the gold standard (reference measure, “label”), and how are they assessed?
- MetRQ3: What types of DHTs are used to collect data, and what modalities are collected?
- MetRQ4: What are the characteristics of data collection (e.g., sampling frequency, follow-up duration)?
- MetRQ5: Which techniques and machine learning algorithms are used in analyzing data from DHTs for the screening or diagnosis of ADRD?

Evaluation questions are related to the results of the research:

- EvaRQ1: How accurate and reliable are passive DHTs in screening or diagnosing ADRD in non-clinical settings?
- EvaRQ2: What specific modalities of the used DHTs are most informative of ADRD?

Implementation questions focus on the discussion of the applicability of such DHTs in clinical settings:

- ImpRQ1: How do individuals perceive and interact with DHTs, and what design features and user interfaces relate to adherence and acceptance?
- ImpRQ2: How can data privacy and security be ensured while enabling effective screening and diagnosis?
- ImpRQ3: How do lifestyle factors and environmental conditions affect the quality of data collected by DHTs?
- ImpRQ4: Does the use of DHTs in non-clinical settings impact the timing and accuracy of ADRD screening and diagnosis?

## Supplementary information

Supplementary Information

## Data Availability

This study does not involve the generation or analysis of new primary datasets. All data extracted from the included articles—comprising study characteristics, sensor modalities, cognitive outcomes, performance metrics, and quality appraisal scores—are fully provided in the Supplementary Materials accompanying this manuscript. All information used in the review is derived from published sources that are cited within the article.
