# Supplementary material for: Passive digital health technologies for Alzheimer’s disease screening and diagnosis: a systematic review
Source: NPJ Digit Med. 2026 Apr 25;9:496. doi: 10.1038/s41746-026-02650-1 (PMC13324020; doi:10.1038/s41746-026-02650-1)
Supplement: Supplementary file 1 — Supplementary Information [file 41746_2026_2650_MOESM1_ESM.pdf]

Supplementary Materials

Passive digital health technologies for Alzheimer's disease screening and diagnosis: a systematic review

Igor Matias[a,b,*], Paweł Prociów[c], Eric J. Daza[d,e], Matthias Kliegel[b], Katarzyna Wac[a]

[a] *Quality of Life Technologies Lab, University of Geneva, Geneva, Switzerland*
[b] *Cognitive Aging Lab, University of Geneva, Geneva, Switzerland*
[c] *DSW University of Lower Silesia, Wrocław, Poland*
[d] *Stats-of-1, Menlo Park, California, United States of America*
[e] *Boehringer Ingelheim Pharmaceuticals Inc., Ridgefield, California, United States of America*

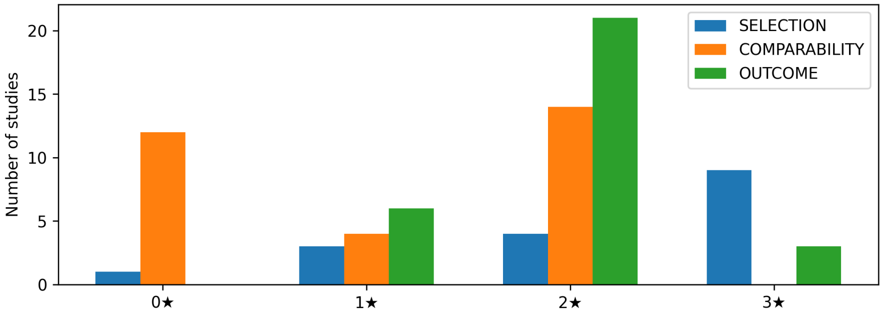

Figure S1: Distribution of domain-level Newcastle-Ottawa scores across studies.

*Corresponding author
Email address: igor.matias@unige.ch (Igor Matias)

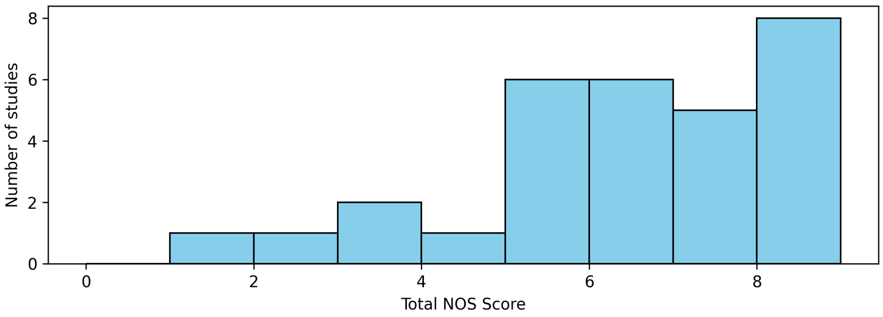

Figure S2: Distribution of total Newcastle-Ottawa scores across studies.

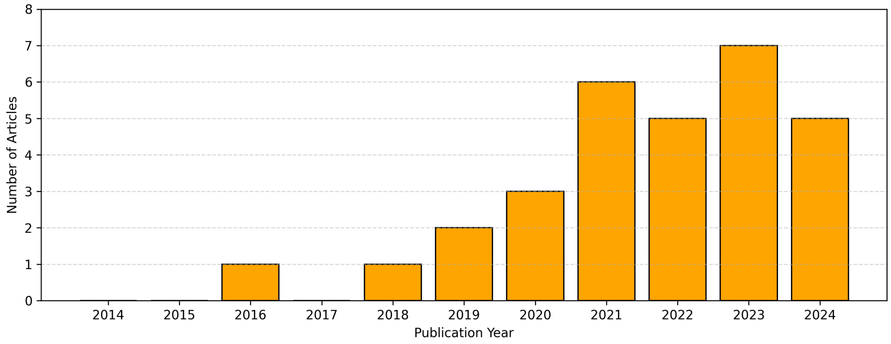

Figure S3: Publication year for the reviewed articles (N = 30). The results for 2024 only represent a portion of the year (until July 9, 52.2% of the year).

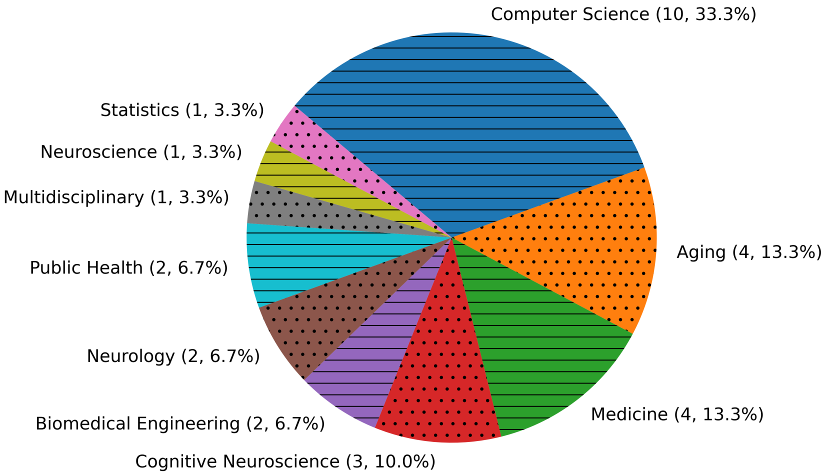

Figure S4: Reviewed publications by field (N = 30).

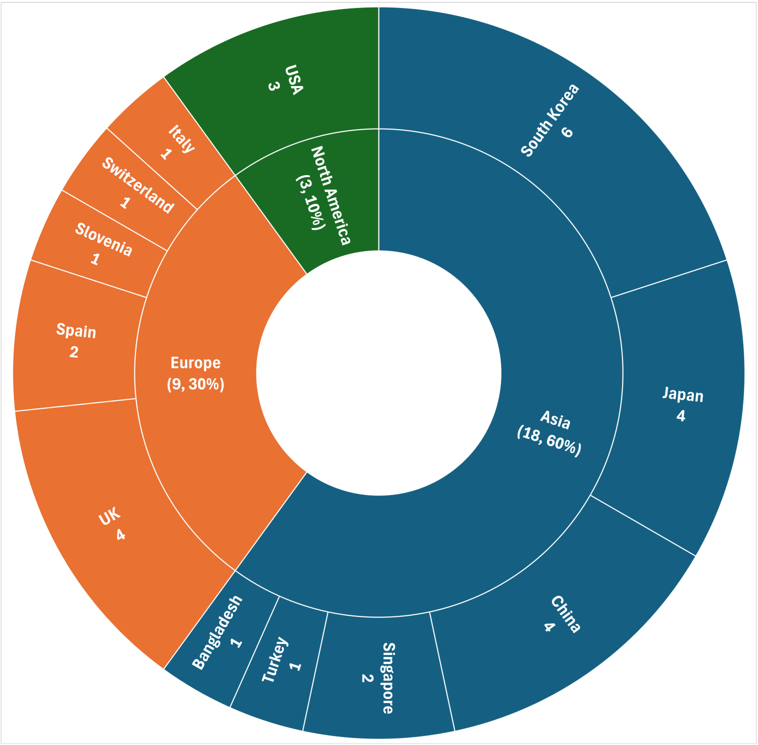

Figure S5: Research group location by continent and country (N = 30).

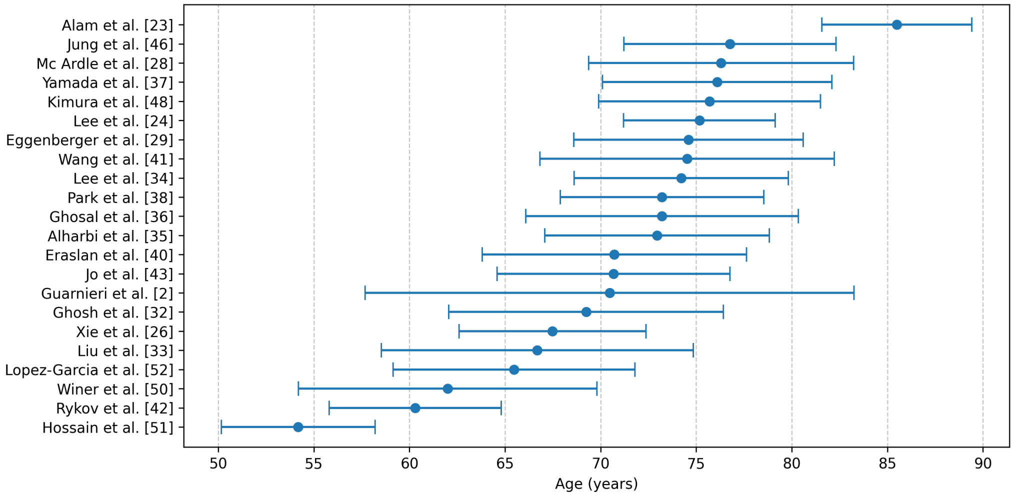

Figure S6: Age distribution of the populations used in the studies who reported mean and SD (22, 73.3%). Sorted from the youngest (bottom) to the oldest (top) population mean. Eight articles did not report this information. References on the Y axis are according to the article's references list.

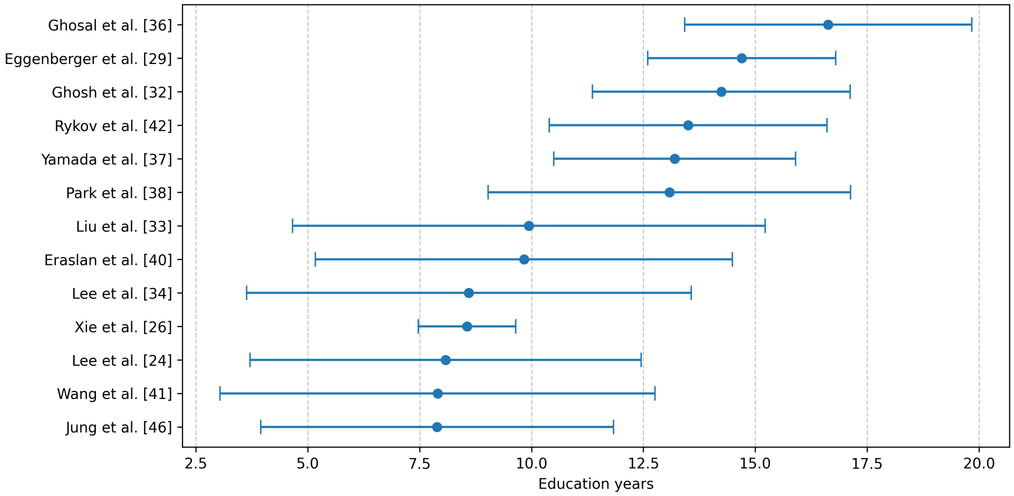

Figure S7: Education years distribution of the populations in the studies that reported mean and standard deviation (43.3%). Sorted from the lowest (bottom) to the highest (top) education years mean. Seventeen studies did not report this information or reported it in a different format or metric and are omitted here. References on the Y axis are according to the article's references list.

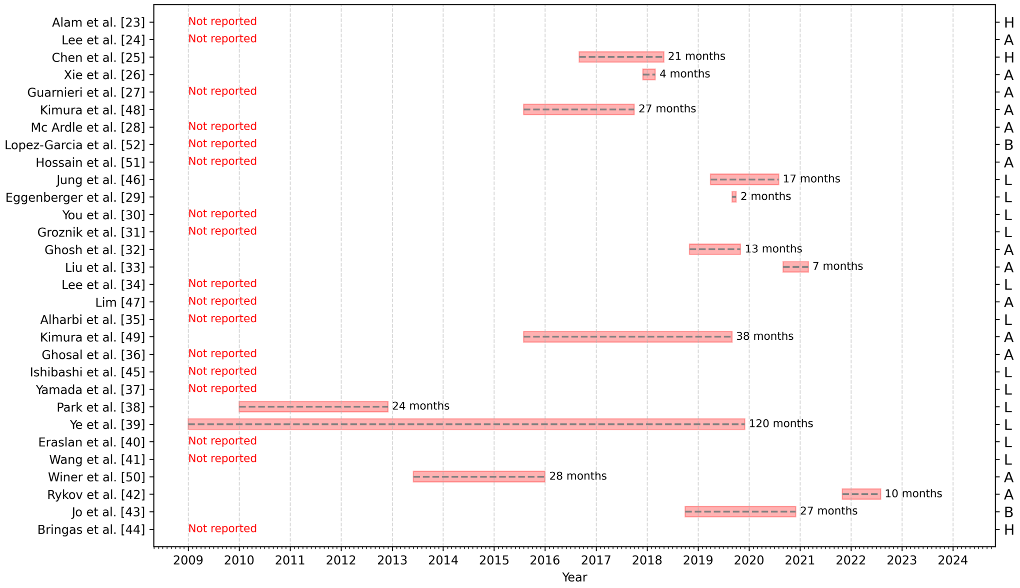

Figure S8: Overall study duration and place of data acquisition (right axis) of the reviewed articles. A stands for anywhere, B for in bed, H for at home, and L for in the lab. References on the Y axis are according to the article's references list.

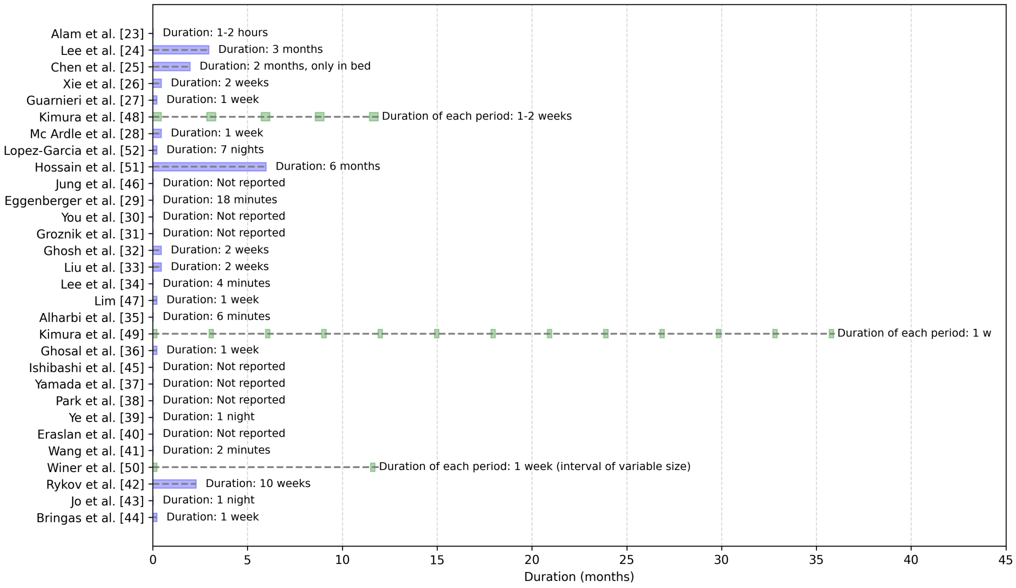

Figure S9: Observation period, duration of data collection, and follow-up duration of the reviewed articles. Purple/full and green/interrupted boxes represent studies with uninterrupted and interrupted data collection, respectively. References on the Y axis are according to the article's references list.

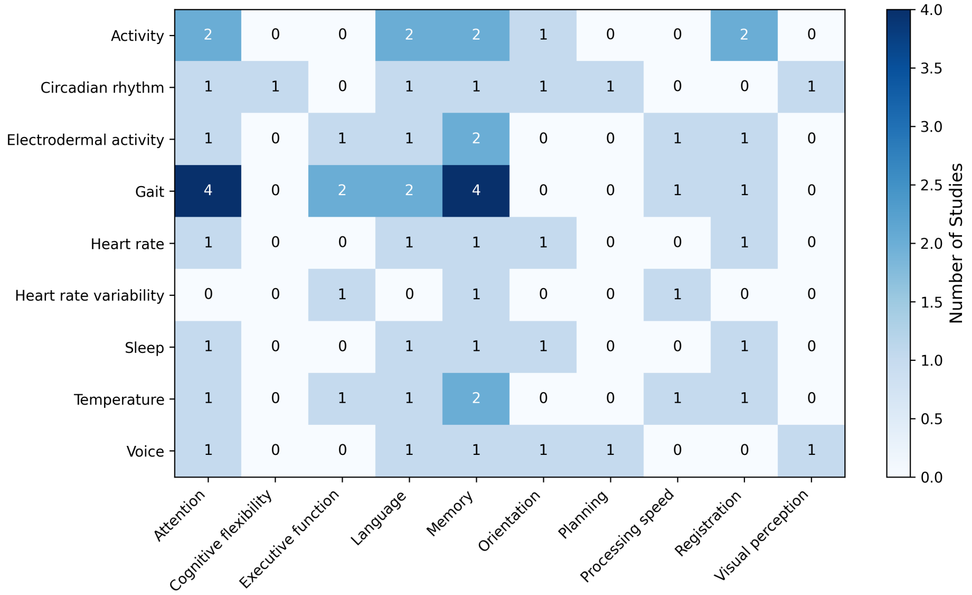

Figure S10: Cross-tabulation of DHT modalities and cognitive domains assessed across the nine reviewed studies that used metrics of cognitive decline as gold standard. Numbers indicate how many studies used a given modality for each cognitive construct.

Table S1: Newcastle–Ottawa Scale quality evaluation for all included studies. The table reports domain-level star ratings for each study under the Selection, Comparability, and Outcome domains according to the corresponding cohort or case–control versions. Total scores range from 0 to 9 stars. These ratings reflect the methodological rigor of participant recruitment and diagnostic ascertainment procedures, independently of the digital technology evaluated in each study. References are according to the article's references list.

**Reference of reviewed study**	**Version**	**Selection**	**Comparability**	**Outcome**	**Total (% of maximum 9)**
Alam et al. [23]	Case-control	4	0	2	6 (66.7%)
Lee et al. [24]	Case-control	2	0	1	3 (33.3%)
Chen et al. [25]	Case-control	1	0	2	3 (33.3%)
Xie et al. [26]	Case-control	4	2	2	8 (88.9%)
Guarnieri et al. [27]	Case-control	3	0	2	5 (55.6%)
Kimura et al. [48]	Cohort	3	2	2	7 (77.8%)
Mc Ardle et al. [28]	Case-control	2	1	2	5 (55.6%)
Lopez-Garcia et al. [52]	Cohort	4	2	2	8 (88.9%)
Hossain et al. [51]	Cohort	4	0	3	7 (77.8%)
Jung et al. [46]	Cohort	4	0	1	5 (55.6%)
Eggenberger et al. [29]	Case-control	3	1	2	6 (66.7%)
You et al. [30]	Case-control	3	0	2	5 (55.6%)

Continued on next page

Table S1 (continued)

**Reference of reviewed study**	**Version**	**Selection**	**Comparability**	**Outcome**	**Total (% of maximum 9)**
Groznik et al. [31]	Case-control	4	1	2	7 (77.8%)
Ghosh et al. [32]	Case-control	4	2	2	8 (88.9%)
Liu et al. [33]	Case-control	4	0	2	6 (66.7%)
Lee et al. [34]	Case-control	3	2	2	7 (77.8%)
Lim [47]	Cohort	4	0	2	6 (66.7%)
Alharbi et al. [35]	Case-control	2	2	1	5 (55.6%)
Kimura et al. [49]	Cohort	4	2	2	8 (88.9%)
Ghosal et al. [36]	Case-control	1	2	1	4 (44.4%)
Ishibashi et al. [45]	Cohort	0	0	1	1 (11.1%)
Yamada et al. [37]	Case-control	2	2	2	6 (66.7%)
Park et al. [38]	Case-control	4	2	2	8 (88.9%)
Ye et al. [39]	Case-control	1	0	1	2 (22.2%)
Eraslan et al. [40]	Case-control	3	2	3	8 (88.9%)
Wang et al. [41]	Case-control	3	1	2	6 (66.7%)
Winer et al. [50]	Cohort	4	2	3	9 (100%)
Rykov et al. [42]	Case-control	4	2	2	8 (88.9%)
Jo et al. [43]	Case-control	3	2	2	7 (77.8%)
Bringas et al. [44]	Case-control	3	0	2	5 (55.6%)

Table S2: Year of publication of each reviewed article, type, field, and name of the venue, and its quartile or rank (in the specified field, matching year of publication if available), and h-index. Quartile information obtained through Scimago Journal Rank, and conference ranking through the CORE portal. NA stands for Not Applicable. References are according to the article's references list.

**Reference of reviewed study**	**Year of publication**	**Type of venue**	**Field of venue**	**Venue**	**Quartile / Rank**	**H-index**
Alam et al. [23]	2016	Conference	Computer Science	IEEE First Conference on Connected Health: Applications, Systems and Engineering Technologies	Not ranked	NA
Lee et al. [24]	2018	Conference	Computer Science	International conference on electronics information and communications	Not ranked	NA
Chen et al. [25]	2019	Conference	Computer Science	IEEE International Conference on Big Data	Rank B	NA
Xie et al. [26]	2019	Journal	Aging	Frontiers in Aging Neuroscience	Quartile 1	120

Continued on next page

Table S2 (continued)

Reference of reviewed study	Year of publication	Type of venue	Field of venue	Venue	Quartile / Rank	H-index
Guarnieri et al. [27]	2020	Journal	Medicine	Journal of Alzheimer's Disease	Quartile 1	177
Kimura et al. [48]	2020	Journal	Medicine	JAMA Network Open	Quartile 1	128
Mc Ardle et al. [28]	2020	Journal	Aging	Gerontology	Quartile 2	113
Lopez-Garcia et al. [52]	2021	Journal	Public Health	Frontiers in Public Health	Quartile 1	101
Hossain et al. [51]	2021	Journal	Biomedical Engineering	Journal of Healthcare Engineering	Quartile 2	57
Jung et al. [46]	2021	Journal	Computer Science	IEEE Journal of Biomedical and Health Informatics	Quartile 1	156
Eggenberger et al. [29]	2021	Journal	Aging	Frontiers in Aging Neuroscience	Quartile 1	120
You et al. [30]	2021	Conference	Computer Science	IEEE International Conference on E-health networking, application and services	Rank C	NA

Continued on next page

Table S2 (continued)

Reference of reviewed study	Year of publication	Type of venue	Field of venue	Venue	Quartile / Rank	H-index
Groznik et al. [31]	2021	Conference	Computer Science	IEEE International Conference on Biomedical and Health Informatics	Not ranked	NA
Ghosh et al. [32]	2022	Journal	Multidisciplinary	Scientific Reports	Quartile 1	315
Liu et al. [33]	2022	Journal	Public Health	Frontiers in Public Health	Quartile 1	101
Lee et al. [34]	2022	Journal	Cognitive Neuroscience	Alzheimer's Research and Therapy	Quartile 1	97
Lim [47]	2022	Journal	Computer Science	Digital Health	Quartile 2	30
Alharbi et al. [35]	2022	Journal	Biomedical Engineering	IEEE Journal of Translational Engineering in Health and Medicine	Quartile 2	41
Kimura et al. [49]	2023	Journal	Cognitive Neuroscience	Alzheimer's Research and Therapy	Quartile 1	97
Ghosal et al. [36]	2023	Journal	Statistics	Biostatistics	Quartile 1	93

Continued on next page

Table S2 (continued)

Reference of reviewed study	Year of publication	Type of venue	Field of venue	Venue	Quartile / Rank	H-index
Ishibashi et al. [45]	2023	Conference	Computer Science	IEEE International Conference on Systems, Man, and Cybernetics (SMC)	Rank B	NA
Yamada et al. [37]	2023	Conference	Computer Science	IEEE International Conference on Digital Health	Not ranked	NA
Park et al. [38]	2023	Journal	Neurology	Neurology	Quartile 1	411
Ye et al. [39]	2023	Journal	Neurology	Sleep	Quartile 1	239
Eraslan et al. [40]	2023	Journal	Neuroscience	Brain and Behavior	Quartile 2	61
Wang et al. [41]	2024	Journal	Aging	Frontiers in Aging Neuroscience	Quartile 2	120
Winer et al. [50]	2024	Journal	Cognitive Neuroscience	Alzheimer's Research and Therapy	Quartile 1	97
Rykov et al. [42]	2024	Journal	Medicine	BMC Medicine	Quartile 1	186
Jo et al. [43]	2024	Journal	Medicine	Dementia and Neurocognitive Disorder	Not ranked	NA
Bringas et al. [44]	2024	Journal	Computer Science	IEEE Journal of Biomedical and Health Informatics	Quartile 1	156

Table S3: Main location of the research team and main objective and contribution of the reviewed research manuscripts. References are according to the article's references list.

**Reference of reviewed study**	**Main location**	**Objective and scientific contribution**
Alam et al. [23]	United States of America	Discriminate MCI patients from healthy controls using physiological sensors and smart home technologies.
Lee et al. [24]	South Korea	Discriminate MCI patients from healthy controls using fitness tracker data.
Chen et al. [25]	Singapore	Discriminate MCI patients from healthy controls using in-home sensors.
Xie et al. [26]	China	Use an inertial-sensor-based wearable collecting daily gait data to see if daily gait deficit was quantitatively associated with cognition.
Guarnieri et al. [27]	Italy	Use wearable-collected sleep and circadian rhythm data to classify people as healthy, MCI or AD.
Kimura et al. [48]	Japan	Use wearable sensors to investigate whether modifiable lifestyle factors are linked to cortical amyloid burden and cerebral metabolism in MCI subjects.
Mc Ardle et al. [28]	United Kingdom	Describe differences in volume, pattern, and variability of habitual activity between people with probable MCI and probable dementia, using a wearable activity monitor.

Continued on next page

Table S3 (continued)

Reference of reviewed study	Main location	Objective and scientific contribution
Lopez-Garcia et al. [52]	Spain	Test whether total sleep time, measured by actigraphy during 1 week, was related to CFS AD core biomarkers in elderly individuals.
Hossain et al. [51]	Bangladesh	Mobile-app keystroke and smartwatch physical data for classification according to MMSE score (normal, mild, moderate, and severe impairment).
Jung et al. [46]	South Korea	Use temporal gait parameters to classify cognitive impairment in the elderly.
Eggenberger et al. [29]	Switzerland	Investigate the association of single-point body temperature measures with cognitive performance in cognitively healthy older adults and in those with MCI.
You et al. [30]	China	Skeleton gait features to distinguish between cognitively healthy and MCI/AD.
Groznik et al. [31]	Slovenia	Investigate the differences in eye movement during reading between people with MCI and healthy controls for automatic detection of MCI.
Ghosh et al. [32]	United Kingdom	Discriminate AD patients from controls by using GPS spatial metrics.
Liu et al. [33]	China	Differentiate MCI from healthy controls using awake and sleeping circadian rhythm.
Lee et al. [34]	South Korea	Determine the optimal electrode configuration for wearable EEG devices to diagnose MCI.

Continued on next page

Table S3 (continued)

Reference of reviewed study	Main location	Objective and scientific contribution
Lim [47]	South Korea	Classify between high and low risk AD individuals with electrodermal activity, acceleration, and temperature wearable sensor.
Alharbi et al. [35]	United Kingdom	Use off-the-shelf heart rate variability monitor devices to distinguish between healthy and MCI.
Kimura et al. [49]	Japan	Predict PET amyloid positivity using wearable data.
Ghosal et al. [36]	United States of America	Capture the distributional nature of wearable data for MCI classification.
Ishibashi et al. [45]	Japan	Dementia diagnosis using gait data.
Yamada et al. [37]	Japan	Voice data for detecting subtle cognitive impairment.
Park et al. [38]	South Korea	Use gait features to classify older adults as cognitively declined or cognitively normal.
Ye et al. [39]	United States of America	Differentiate cognitively normal from MCI and dementia of different etiologies using EEG sleep data.
Eraslan et al. [40]	Turkey	Examine differences in eye movement during scanning of faces between cognitively healthy, AD, and aMCI.
Wang et al. [41]	China	Explore new gait indicators that can distinguish patients with cognitive impairment from normal older adults.
Winer et al. [50]	United Kingdom	Determine whether accelerometer-derived metrics of 24-h activity patterns are associated with longitudinal cognitive test performance and risk of incident AD.

Continued on next page

Table S3 (continued)

Reference of reviewed study	Main location	Objective and scientific contribution
Rykov et al. [42]	Singapore	Using wearable physiological data to predict inter- and intra-individual changes in cognitive function.
Jo et al. [43]	South Korea	Investigate whether the accumulation of Amyloid beta is related to certain characteristics of sleep in the elderly with subjective cognitive decline.
Bringas et al. [44]	Spain	Identify the AD stage based on gait data.

Table S4: Education years, inclusion and exclusion criteria, and APOE4 state of the population used in each study reviewed. NR stands for not reported and indicates that information was not found in the respective article. References are according to the article's references list.

**Reference of reviewed study**	**Education years**	**Inclusion criteria**	**Exclusion criteria**	**APOE4**
Alam et al. [23]	NR	Age $\geq$ 65	Living with another person	NR
Lee et al. [24]	M = 8.08, SD = 4.37	NR	NR	
Chen et al. [25]	NR	Age $\geq$ 65, able to function independently within the community	NR	NR
Xie et al. [26]	M = 8.56, SD = 1.09	Ages $\geq$ 55, able to walk independently without any gait assistance	Any neurologic disorder and other systematic disease that would likely contribute to cognitive and or motor deficits, active rheumatic and orthopedic disease, use of neuroleptics or benzodiazepines, psychiatric comorbidity	NR

Continued on next page

Table S4 (continued)

Reference of reviewed study	Education years	Inclusion criteria	Exclusion criteria	APOE4
Guarnieri et al. [27]	NR	Age $\geq$ 40, Clinical Dementia rating scale score of 2 or lower	Concomitant unstable medical conditions, other neurological or psychiatric diseases, stroke reported at the onset of cognitive symptoms, relevant vascular load detectable on the brain MRI (Fazekas score above 2), use of neuropsychiatric drugs	NR
Kimura et al. [48]	Mdn = 12.0, IQR = 9-12	Age $\geq$ 65, without dementia, independent function in activities of daily living	NR	NR
Mc Ardle et al. [28]	NR	Age $\geq$ 60, able to walk for 2 minutes	Drug-induced or vascular parkinsonism, co-existing neurological conditions or movement disorders, severe mental illness, evidence of stroke affecting motor function, poor command of English	NR

Continued on next page

Table S4 (continued)

**Reference of reviewed study**	**Education years**	**Inclusion criteria**	**Exclusion criteria**	**APOE4**
Lopez-Garcia et al. [52]	34.5% tertiary, 38.8% secondary, 26.7% primary	Age ≥ 55, cognitively unimpaired, with a Clinical Dementia Rating of 0	NR	NR
Hossain et al. [51]	NR	Age between 50 and 65	NR	
Jung et al. [46]	M = 7.89, SD = 3.94	Age ≥ 65, able to walk 10 meters without any walking aids or assistance from others	Paraparesis, hemiparesis, dyskinetic cerebral palsy, psychogenic movement disorders, structural abnormalities in both legs at the time of recruiting	NR
Eggenberger et al. [29]	M = 14.7, SD = 2.1	Age ≥ 65, be able to walk at least 8 min with or without walking aids, in no more than minimal need for care or treatment	Previous diagnosis of dementia or recent head injury	14.4% positive
You et al. [30]	NR	Age > 46, able to walk independently	NR	NR
Groznik et al. [31]	NR	Age ≥ 40	Uncorrected visual impairments, concomitant neurological diseases, drug or alcohol abuse	30.7% positive

Continued on next page

Table S4 (continued)

**Reference of reviewed study**	**Education years**	**Inclusion criteria**	**Exclusion criteria**	**APOE4**
Ghosh et al. [32]	M = 14.24, SD = 2.88	Age between 50 and 80, residing at home	Previous history of alcohol or substance abuse, presence of a psychiatric condition	NR
Liu et al. [33]	M = 9.94, SD = 5.28	Age between 60 and 75, diagnosis of MCI according to the 2018 Chinese Guidelines for the Diagnosis and Treatment of Dementia and Cognitive Impriament, having normal activities of daily living, able to self-care	Geriatric Depression Scale score above 8, a history of depression. brain tumors, Parkinson's disease, other medical conditions affecting brain or cognitive function, history of acute illness within 3 months, diagnosis of active epilepsy, secondary sleep-wake rhythm disorder	NR
Lee et al. [34]	M = 8.60, SD = 4.97	NR	Being pregnant, having any of other neurological or comorbid disorders, organic brain damage, or impairments in sensory or motor functions	NR
Lim [47]	NR	Age $\geq$ 65, capable of communication, K-MMSE $\geq$ 20, capable of performing activities of daily living	NR	NR

Continued on next page

Table S4 (continued)

Reference of reviewed study	Education years	Inclusion criteria	Exclusion criteria	APOE4
Alharbi et al. [35]	NR	Age between 60 and 90, diagnosed with MCI	Diagnosis of a neurological condition, MMSE < 24, current alcohol or substance misuse, history of cardiovascular conditions	NR
Kimura et al. [49]	Mdn = 12.0, IQR = 9-12	Age ≥ 65, without dementia, independent function in activities of daily living	NR	13.9% positive
Ghosal et al. [36]	M = 16.63, SD = 3.21	NR	NS	NR
Ishibashi et al. [45]	NR	NR	NR	NR
Yamada et al. [37]	M = 13.2, SD = 2.7	NR	NR	NR
Park et al. [38]	M = 13.08, SD = 4.05	Age ≥ 60, living in one of 30 villages and towns across South Korea	History of stroke, Parkinson's disease or other movement disorders, history of unilateral knee, bilateral knee, or hip arthroplasty knee, hip, or ankle joint surgery, spinal diseases	NR

Continued on next page

Table S4 (continued)

Reference of reviewed study	Education years	Inclusion criteria	Exclusion criteria	APOE4
Ye et al. [39]	NR	Having undergone polysomnogram(s) for clinical purposes in the Sleep Laboratory at Massachusetts General Hospital from 2009 to 2019	NR	NR
Eraslan et al. [40]	M = 9.83, SD = 4.66	Unaided normal vision or wearing corrective lenses	Hearing impairment that might impede effective communication with investigators	NR
Wang et al. [41]	M = 7.90, SD = 4.86	Age $\geq$ 60, able to walk independently for 10 minutes without assistance, without severe physical disease	Neurological or skeletal muscle diseases that may cause gait abnormalities, several mental diseases, severe visual impairment, hearing impairment, obvious white matter lesions	NR
Winer et al. [50]	NR	Age between 40 and 70	None	NR
Rykov et al. [42]	M = 13.5, SD = 3.1	Age between 50 and 70, diagnosed with amnestic MCI using the Petersen's or the NIA-AA criteria, clinical dementia rating score of 0.5, MMSE > 24, education > 6 years, basic digital proficiency	Significant hearing or visual impairment, systemic, neurological, or psychiatric illness, participation in any other clinical trial in the preceding 12 weeks	NR

Continued on next page

Table S4 (continued)

**Reference of reviewed study**	**Education years**	**Inclusion criteria**	**Exclusion criteria**	**APOE4**
Jo et al. [43]	All at least elementary school graduation	Age $\geq$ 60, minimum elementary school graduation, normal activities of daily living, Seoul verbal learning test delayed recall score between -1.5 and 0 SD	Vitamin B9 or B12 deficiency, syphilis, thyroid function abnormality, anxiety disorder, somatic symptom disorder, uncontrolled depression, schizophrenia, alcohol and substance dependence, other non-AD causes of cognitive impairment	18.4% positive
Bringas et al. [44]	NR	Global Deterioration Scale $\geq$ 2	Global Deterioration Scale of 1	NR

References	Not shared	Upon request	Publicly available
Alam et al. [23]	X		
Lee et al. [24]	X		
Chen et al. [25]	X		
Xie et al. [26]		X	
Guarnieri et al. [27]	X		
Kimura et al. [48]	X		
Mc Ardle et al. [28]	X		
Lopez-Garcia et al. [52]		X	
Hossain et al. [51]		X	
Jung et al. [46]	X		
Eggenberger et al. [29]		X	
You et al. [30]	X		
Groznik et al. [31]	X		
Ghosh et al. [32]		X	
Liu et al. [33]		X	
Lee et al. [34]	X		
Lim [47]	X		
Alharbi et al. [35]	X		
Kimura et al. [49]	X		
Ghosal et al. [36]			X (code only)
Ishibashi et al. [45]	X		
Yamada et al. [37]	X		
Park et al. [38]		X	
Ye et al. [39]	X		
Eraslan et al. [40]		X	
Wang et al. [41]		X	
Winer et al. [50] *			X
Rykov et al. [42]		X	
Jo et al. [43] *	X		
Bringas et al. [44]	X		

Table S5: Data-sharing policy of each of the reviewed studies. Two studies used datasets from previous studies and are marked with a *. References are according to the article's references list.

Ref.	Category	Outcome description
23	Identified stage of ADRD or healthy	Prior categorization between: CH, MCI, cognitively impaired
24	Identified stage of ADRD or healthy	Prior categorization between: CH, MCI
25	Identified stage of ADRD or healthy	Prior categorization between: CH, MCI
26	Metrics of cognitive decline	Executive function, memory (long and short term), language, attention
27	Identified stage of ADRD or healthy	Prior categorization between: CH, AMCI, AD
48	Clinical ADRD biomarkers	Cortical Amyloid Burden, Cerebral Glucose Metabolism
28	Identified stage of ADRD or healthy	Prior categorization between: CH, AD (and dementia with Lewy bodies, Parkinson's disease dementia, not focused on by this research)
52	Clinical ADRD biomarkers	AB42, AB40, Phosphorylated-tau-181, total-tau
51	Metrics of cognitive decline	Standardized Mini-Mental State Examination
46	Metrics of cognitive decline	MMSE to classify the participants as CH, low-risk or high-risk of AD
29	Identified stage of ADRD or healthy	Prior categorization between: CH, MCI
30	Identified stage of ADRD or healthy	Prior categorization between: CH, MCI, AD
31	Identified stage of ADRD or healthy	Prior categorization between: CH, MCI, AD
32	Identified stage of ADRD or healthy	Prior categorization between: CH, AD
33	Identified stage of ADRD or healthy	Prior categorization between: CH, MCI
34	Identified stage of ADRD or healthy	Prior categorization between: CH, MCI
47	Metrics of cognitive decline	Korean MMSE
35	Identified stage of ADRD or healthy	Prior categorization between: CH, MCI
49	Clinical ADRD biomarkers	PET brain amyloid
36	Metrics of cognitive decline	Attention, Verbal Memory, Executive Function
45	Metrics of cognitive decline	Classification as normal, low, and high cognitive abilities using the Digit Symbol Substitution Test
37	Metrics of cognitive decline	ECog scale: memory, language, visual perception, planning, organization, divided attention
38	Identified stage of ADRD or healthy	Prior categorization between: CH, MCI, AD (and dementia with Lewy bodies, not focused on by this research)
39	Identified stage of ADRD or healthy	Prior categorization between: CH, MCI, dementia of different etiologies
40	Identified stage of ADRD or healthy	Prior categorization between: CH, MCI, AD
41	Identified stage of ADRD or healthy	Prior categorization between: CH, MCI, dementia
50	Metrics of cognitive decline	Symbol substitution test, trail making numbers, trail making numbers letters, numeric memory, fluid intelligence
42	Metrics of cognitive decline	Executive function, processing speed, immediate and delayed memory
43	Clinical ADRD biomarkers	Amyloyd accumulation as measured with PET scan
44	Identified stage of ADRD or healthy	Prior categorization between: early, moderate, severe stages of AD

Table S6: Outcomes used as the ground truth (reference measure) in the reviewed research. References are according to the article's references list.

Table S7: DHTs used to collect data in the reviewed studies, their type, model, and features extracted from them. References are according to the article's references list.

Ref.	Type(s) of sensor used	Device(s) used	Features extracted
23	Accelerometer, smart home sensors, EDA sensor, PPG	Smart home: Pogo-Plus; Wearable: Empatica E4	EDA, HRV, smart home task: activity ability, sequencing, interruptions, duration
24	Accelerometer, PPG, altimeter	Fitbit Charge 2	Step count, distance walked, floors climbed, calories burned, asleep, restless, awake, sedentary duration, lightly active, fairly active, highly active
25	Bed sensor	NR	Sleep states
26	Micro-Electro-Mechanical System sensors fixed under the shoe heel bottom	Purpose-made	Steps, walking velocity, stride length, stride time, cadence, stride time variability
27	Accelerometer	Fitbit Flex	Sleep: total sleep time, WASO, sleep efficiency, sleep regularity index. Circadian: acrophase, nadir, amplitude, mesor, period, circadian quotient, rhythm quotient
48	Accelerometer, PPG, temperature sensor, microphone	Silmee W20 with attached microphone	Walking steps, conversation time, TST, WASO, sleep efficiency, walking time count, nap time

Continued on next page

Table S7 (continued)

Ref.	Type(s) of sensor used	Device(s) used	Features extracted
[28]	Accelerometer	Axivity AX3	Total steps per bout, bout length, total number of bouts, total walk time, total steps, alpha, variability of bout length
[52]	Accelerometer	Xiaomi Mi Band 2	Total sleep time
[51]	Accelerometer, PPG	NR	Absolute energy, quality sleeping time, total steps, sitting time, daily HR average, total distance, cycling time
[46]	Accelerometer, gyroscope	Xsens MVN	Gait sequence feature for: initial double-limb support time, single-limb support time, terminal double-limb support time, stance time, swing time, step time, stride time
[29]	Skin temperature sensor	MSR thermistors	Skin temperature at: right lateral rib cage, right scapula
[30]	Motion sensor camera	Microsoft Kinect V2	Skeleton sequences
[31]	Eye tracker	90 HZ Tobii 4C	Forward and backward saccade distance, amount of variation in backward saccade distances, forward and backward saccade speed, duration of fixations, amount of variation in the fixation duration, ratio between forward and backward saccades, number of fixations per second
[32]	GPS tracker	Trackershop Pro Pod 5	Street name, speed, battery level, distance traveled, signal accuracy, latitude, and longitude

Continued on next page

Table S7 (continued)

Ref.	Type(s) of sensor used	Device(s) used	Features extracted
[33]	Accelerometer, PPG, oximeter	W180 Fitfaith Technologies China	Daytime physical activity, sedentary time, night-time HR, HRV, respiratory rate, oxygen saturation
[34]	EEG	SynAmps amplifier from 32 scalp electrodes	Absolute power spectrum density, relative power spectrum density, differential asymmetry, rational asymmetry, phase-amplitude coupling, Shannon entropy, Hjorth parameters, Lyapunov exponent, hurst exponent, Kolmogorov complexity
[47]	Accelerometer, skin temperature, EDA sensor	NR	EDA vertical amplitude, EDA horizontal duration, acceleration, temperature
[35]	PPG (finger worn)	CorSense	Mean respiratory rate, ln(SDNN), ln(RMSSD), ln(HF)
[49]	PPG, Accelerometer, microphone	Silmee W20	Steps, light PA, moderate-to-vigorous PA, sedentary behavior, TST, sleep efficiency, awakening time count, nap time, nap efficiency, WASO during nap, HR, conversation time, ActiveScale (hours with at least 250 steps)
[36]	Accelerometer	GT3x+	Four L-moments of Stride regularity, step velocity, and cadence
[45]	Micro-Doppler radar	ILT Office BSS-10	Gait speed over time
[37]	Microphone	Apple Watch Series 6	42 features related to the voice spectrum, pitch, formant, voice quality

Continued on next page

Table S7 (continued)

Ref.	Type(s) of sensor used	Device(s) used	Features extracted
[38]	Accelerometer, gyroscope	Fitmeter	Cadence, step time, gait speed, step length, step time variability, step time asymmetry, gait variability
[39]	EEG	NR	Six EEG channels (F3-M2, F4-M1, C3-M2, C4-M1, O1-M2, O2-M1). Macro-structure sleep features: total resting time, total sleep time, duration of sleep stages, percent of the time in each stage, sleep efficiency index, sleep onset latency, WASO, REM latency, NA, number of stage shifts to N1 from NREM/REM, sleep fragmentation index. Micro-structural sleep features: line length, kurtosis, sample entropy, minimum, maximum, mean, and SD across 2-second sub-epochs within each 30-second epoch.
[40]	Infrared eye tracking system	SR Research EyeLink 1000 Plus	Number of fixations, mean fixation duration, first fixation duration
[41]	Accelerometer, Gyroscope, Magnetometer, Barometer	APDM Mobility Lab	Gait speed, stride length, turn velocity, cadence, double support, lateral step variability, stance, swing
[50]	Accelerometer	AX3	Mesor (rhythm-adjusted mean activity), amplitude, intraday variability, day-to-day stability, activity level during the least active 5 h, activity level during the most active 10 h

Continued on next page

Table S7 (continued)

Ref.	Type(s) of sensor used	Device(s) used	Features extracted
[42]	Accelerometer, skin temperature, EDA sensor, blood volume pulse, PPG	Empatica E-4	Electrodermal activity, heart rate, heart rate variability, inter-beat interval, skin temperature
[43]	PPG	Fitbit Alta HR	Total sleep time, total time spent in bed, time spent awake in bed, number of times awakened during sleep, total REM sleep time, total time in light sleep, total time in slow-wave sleep
[44]	Mobile phone accelerometer	Android mobile phone accelerometer	Raw accelerometer signal

Table S8: Study duration, and place and frequency of data acquisition of the reviewed articles. References are according to the article's references list.

**Ref.**	**Overall study duration**	**Place**	**Data acquisition frequency**
[23]	NR	Home	Once
[24]	NR	Anywhere	Continuously
[25]	September 2016 to May 2018	Home	Continuously
[26]	December 2017 to March 2018	Anywhere	Continuously
[27]	NR	Anywhere	Continuously
[48]	August 2015 to October 2017	Anywhere	Every 3 months
[28]	NR	Anywhere	Continuously
[52]	NR	In bed	Every night
[51]	NR	Anywhere	Continuously
[46]	April 2019 to August 2020	In lab	Once
[29]	September to October 2019	In lab	Once
[30]	NR	In lab	Once
[31]	NR	In lab	Once
[32]	November 2018 to November 2019	Anywhere	Continuously
[33]	September 2020 to March 2021	Anywhere	Continuously
[34]	NR	In lab	Once
[47]	November 2020 to February 2021	Anywhere	Continuously
[35]	NR	In lab	Continuously
[49]	August 2015 to September 2019	Anywhere	Continuously
[36]	NR	Anywhere	Continuously
[45]	NR	In lab	Continuously

Continued on next page

Table S8 (continued)

Ref.	Overall study duration	Place	Data acquisition frequency
37	NR	In lab	Once
38	2010 to 2012	In lab	Once
39	2009 to 2019	In lab	Once
40	NR	In lab	Once
41	NR	In lab	Once
50	June 2013 to January 2016	Anywhere	Continuously
42	November 2021 to August 2022	Anywhere	Every night
43	October 2018 to December 2020	In bed	Once
44	NR	Home	Continuously

Table S9: Observation period, duration of data collection, and follow-up duration of the reviewed articles. References are according to the article's references list.

Ref.	Observation period	Duration	Follow-up
23	While performing a series of smart home tasks in a specified order	1 to 2 hours	NA
24	Continuously	3 months	NA
25	In bed only	2 months	NA
26	During normal walk moments	2 weeks	NA
27	Continuously	1 week	NA
48	Continuously, every 3 months in 1 year	1 to 2 weeks	1 year
28	Continuously	1 week	NA
52	Only at night	7 nights	NA
51	Continuously	6 months	NA

Continued on next page

Table S9 (continued)

Ref.	Observation period	Duration	Follow-up
[46]	While walking along a 7-meter path	NR	NA
[29]	While either stopped or walking at a natural pace a 20-meter path	18 minutes	NA
[30]	While walking at natural pace a 10-meter path	NR	NA
[31]	While reading from a screen 70 centimeters away	NR	NA
[32]	Continuously	2 weeks	NA
[33]	Continuously for PA, only night for HR-related features	2 weeks	NA
[34]	In lab, with eyes closed	4 minutes	NA
[47]	Continuously	1 week	NA
[35]	08:30 AM to 12:00 PM	6 minutes	NA
[49]	Continuously except when bathing	1 week every 3 months	3 years
[36]	During identified episodes of sustained walking (longer than 60 seconds)	1 week	
[45]	In lab	NR	NA
[37]	Conversation while performing cognitive tasks and while having a daily life chat	Once	NA
[38]	While walking at natural pace a 14-meter path	NR	NA
[39]	In lab	One night	NA
[40]	In lab	NR	NA
[41]	While walking at natural pace a 7-meter path	2 minutes	NA
[50]	Continuously, between two cognitive assessments within a year	1 week	1 year
[42]	During the calmest 5-h night-time window (from 1 to 6 AM)	10 weeks	NA

Continued on next page

Table S9 (continued)

Ref.	Observation period	Duration	Follow-up
[43]	At night	One night	NA
[44]	Continuously	1 week	NA

Table S10: Data validity threshold criteria and data imputation strategies reported by the reviewed articles. Only the manuscripts that mentioned at least one of these information are shown. References are according to the article's references list.

Ref.	Data validity threshold	Data imputation method
[25]	Minimum 1 month (50% of the duration)	None
[26]	Minimum 1 hour every day	NR
[28]	Minimum 3 days of data (42.86% of the duration)	NR
[33]	Missing data if 90 minutes of PA missing at least	NR
[34]	5-s epochs with maximal absolute potential value not exceeding 75 microVolts, minimum of 20 epochs per subject (41.67% of the duration)	NR
[39]	NR	Missing sleep stages: 10 k-nearest neighbor imputation
[50]	Minimum 5 days of data (72.43% of the duration), not collected during a daylight saving time shift or the week following it, not having at least 24-h off-wrist period, not having implausible high acceleration values	Using the median of similar time-of-day vector magnitude and intensity distribution data points with 30-2 granularity on different days of the measurement

Continued on next page

Table S10 (continued)

**Ref.**	**Data validity threshold**	**Data imputation method**
[42]	Minimum 50% of HR and temperature, 70% for EDA and PPG, while the number of heartbeats and IBI could not differ from average HR in the same segment by more than 10%. Daily summaries only for days with at least four valid 5-minute samples	Linear interpolation on gaps less than 30 seconds

Table S11: Methodologies adopted by the reviewed articles following a comparison or correlation approach. References are according to the article's references list.

**Ref.**	**Approach**	**Feature selection**	**Method used**	**Adjusting for**	**Evaluation**
[28]	Comparison	NR	ANCOVA	None	P-value, effect size
[40]	Comparison	NR	ANOVA, Bonferroni correction	None	P-value, ANOVA repeated measures mean
[43]	Comparison	NR	Independent samples t-test	Age	P-value, effect size
[23]	Correlation	NR	Pearson correlation	None	P-value, Pearson r
[26]	Correlation	NR	NR	None	Correlation r, p-value
[48]	Correlation	NR	Multiple linear regression	Age, sex, education, APOE4, BMI, vascular risk factors, alcohol consumption, smoking status	Beta, p-value
[52]	Correlation	NR	Pearson correlation	Age, sex, APOE4	Beta, p-value
[29]	Correlation	NR	Spearman correlation, Mann-Whitney test	Age	Spearman r, p-value, AUC

Continued on next page

Table S11 (continued)

Ref.	Approach	Feature selection	Method used	Adjusting for	Evaluation
50	Correlation	Functional PCA (top 4, explaining $\geq$ 90% of the variance)	Linear mixed regression	Age, sex, college education, self-reported general health, BMI, Townsend deprivation index, if in-person or remote assessment	Beta, p-value

Table S12: Methodologies adopted by the reviewed articles following classification or regression approaches, except evaluation metrics. References are according to the article's references list.

Ref.	Approach	Feature selection	Method used	Cross-validation	Adjusting for
[24]	Classification	NR	Artificial Neural Network	6-fold	None
[25]	Classification	NR	Logistic regression	NA	None
[27]	Classification	NR	Decision tree (not specified)	NA	None
[51]	Classification	Wrapper feature	Gradient Boosting, Support Vector Machine	10-fold, with a 5*2 approach	None
[46]	Classification	NR	Long Short Term Memory (ANN)	5-fold	None
[30]	Classification	NR	Long Short Term Memory (ANN)	NR	None
[31]	Classification	NR	Logistic regression, Naive Bayes, eXtreme Gradient Boosting, Random Forest	10-fold	None
[32]	Classification	NR	Logistic regression	Leave one out	None

Continued on next page

Table S12 (continued)

Ref.	Approach	Feature selection	Method used	Cross-validation	Adjusting for
[33]	Classification	Applied Random Forest model to each feature group separately and selected the top 5 features.	Gradient Boosting Decision Tree, eXtreme Gradient Boosting	Leave one out (subject-dependent)	None
[34]	Classification	Fisher's score	Support Vector Machine	Leave-pair-out	None
[47]	Classification	Principal Component Analysis	Deep Neural Network with quantile PCA scaling	34% of the dataset for validation	None
[35]	Classification	NR	Logistic regression	10-fold	Age, sex
[49]	Classification	Boruta method	Support Vector Machine, ElasticNet, Logistic Regression	5-fold with 10 different seeds	Age, sex, education years, BMI
[45]	Classification	NR	Convolutional Neural Networks, Vision Transformers	NA	None
[37]	Classification	Boruta method	Logistic regression, Xgboost, LightGBM, Support Vector Machine, K-nearest neighbors	10x3 nested	Age, sex, education years

Continued on next page

Table S12 (continued)

Ref.	Approach	Feature selection	Method used	Cross-validation	Adjusting for
[38]	Classification	NR	Logistic regression	Bootstrap	Age, sex, education, height, presence of lower limb arthritis
[39]	Classification	ANOVA, Random Forest	Logistic regression, Support Vector Machine, Random Forest	NR	None
[41]	Classification	NR	Multiple linear regression	NA	Age, educational level
[44]	Classification	NR	Convolutional Neural Network using non-continual learning	10-fold	None
[36]	Regression	NR	Linear regression	10-fold	Age, sex
[42]	Regression	Statistical filter based on Pearson and Spearman correlations. Highly correlated with each other were removed.	Elastic net, Random Forest, Extreme Gradient Boosting	Leave one out (subject- and interval-dependent)	None

Table S13: Evaluation metrics used by the reviewed articles following a classification or regression approach. References are according to the article's references list.

**Ref.**	**Approach**	**Accuracy**	**Sensitivity**	**Specificity**	**Precision**	**F1 score**	**AUC**	**Other**
[24]	Classification						X	
[25]	Classification	X	X		X			
[27]	Classification	X	X	X	X			
[51]	Classification	X	X		X	X	X	
[46]	Classification		X		X	X		
[30]	Classification	X	X	X				
[31]	Classification	X					X	Brier score
[32]	Classification		X	X				
[33]	Classification	X	X		X	X	X	
[34]	Classification	X	X	X				
[47]	Classification	X	X	X	X		X	
[35]	Classification	X	X	X				
[49]	Classification		X		X	X	X	
[45]	Classification	X	X		X			F2 score
[37]	Classification	X	X	X		X		
[38]	Classification	X					X	
[39]	Classification						X	AUPRC
[41]	Classification		X	X			X	
[44]	Classification	X	X		X	X		

Continued on next page

Table S13 (continued)

Ref.	Approach	Accuracy	Sensitivity	Specificity	Precision	F1 score	AUC	Other
[36]	Regression							R-squared
[42]	Regression							MAE

Table S14: Evaluation results from research applying a comparison approach. References are according to the article's references list.

Ref.	P-value	Effect size	ANCOVA F	ANOVA mean
[28]	(CH vs dementia) Walking time per day: ≤ 0.001. Steps per day: ≤ 0.001. Bouts per day: ≤ 0.001. Mean bout length: ≤ 0.001. Variability: ≤ 0.001.	(CH vs dementia) Walking time per day: 0.083. Steps per day: 0.110. Bouts per day: 0.083. Mean bout length: 0.138. Variability: 0.123.	(CH vs dementia) Walking time per day: 9.3. Steps per day: 12.7. Bouts per day: 9.3. Man bout length: 16.5. Variability: 14.5.	NA
[40]	Number of fixations: AD vs CH 0.045, AD vs aMCI 0.019	NA	NA	Number of fixations on eyes: AD 3.03 ± 2.26, CH 4.64 ± 2.87. On mouth: AD 1.78 ± 1.65, aMCI 1.42 ± 1.17.
[43]	(Amyloid-negative vs positive) Total time in slow-wave sleep: 0.009.	Amyloid-negative + 10.1 minutes of total time in slow-wave sleep.	NA	NA

Table S15: Evaluation results from research applying a correlation approach. References are according to the article's references list.

Ref.	P-value	Correlation coefficient	Beta	AUC
[23]	Between NCI, MCI, and CI: smart home task interruptions <0.05. Between NCI and MCI: EDA <0.05, HRV <0.05. Between NCI and CI: EDA <0.05, HRV <0.05.	(Pearson) Between NCI, MCI, and CI: smart home task interruptions 0.638. Between NCI and MCI: EDA 0.51, HRV -0.51. Between NCI and CI: EDA -0.58, HRV 0.69.	NA	NA
[26]	Walking velocity: 0.043 with long-term memory; 0.007 with executive function. Stride length: 0.036 with long-term memory; 0.024 with executive function; 0.048 with attention. Stride time variability: 0.038 with long-term memory; 0.022 with executive function; 0.015 with attention.	NA	Walking velocity: 0.256 with long-term memory; 0.49 with executive function. Stride length: 0.322 with long-term memory; 0.366 with executive function; 0.264 with attention. Stride time variability: -0.206 with long-term memory; -0.430 with executive function; -0.247 with attention.	NA

Continued on next page

Table S15 (continued)

Ref.	P-value	Correlation coefficient	Beta	AUC
[48]	With cerebral glucose metabolism: conversation time 0.04; total sleep time <0.001; WASO 0.05; walking time count 0.03.	NA	With cerebral glucose metabolism: conversation time 0.183; total sleep time -0.287; WASO -0.176; walking time count -0.206.	NA
[52]	Total sleep time associated with: t-tau 0.00036; p-tau 0.0072.	NA	Total sleep time associated with: t-tau -0.96; p-tau -0.14	NA
[29]	Right lateral rib cage temp: processing speed 0.006 sitting, 0.002 walking.	Right lateral rib cage temp: processing speed 0.28 sitting, 0.33 walking.	NA	Right lateral rib temp: 0.618 sitting, 0.626 walking. Right scapula temp: 0.558 sitting, 0.551 walking.

Continued on next page

Table S15 (continued)

Ref.	P-value	Correlation coefficient	Beta	AUC
?	(24h rhythm associations, only stat. signif., all *time) With Symbol digit: most active 10h (M10) 0.01, amplitude 0.048, mesor 0.01. With Trails A: interday stability (IS) 0.005, interday variability (IV) 0.001, M10 0.03, amplitude 0.005. With Trails B: IV 0.001. With numeric memory: IV 0.01. With fluid intelligence: IS 0.05.	NA	(24h rhythm associations, only stat. signif., all *time) With Symbol digit: most active 10h (M10) 0.002, amplitude 0.002, mesor 0.004. With Trails A: interday stability (IS) -0.89, interday variability (IV) 0.50, M10 -0.01, amplitude -0.01. With Trails B: IV -0.75. With numeric memory: IV 0.05. With fluid intelligence: IS -0.07.	NA

Table S16: Evaluation results from research applying a classification approach. References are according to the article's references list.

Ref.	Accuracy	Sensitivity	Specificity	Precision	F1 score	AUC	Other
[24]	NA	NA	NA	NA	NA	81.00%	NA
[25]	74.00%	78.60%	NA	78.60%	NA	NA	NA
[27]	89.87%	89.87%	89.87%	89.87%	NA	NA	NA
[51]	Between all classes: gradient boosting 94.80%, SVM 61.50%. Normal: GB 96.60%, SVM 87.80%. Mild: GB 97.70%, SVM 45.50%. Moderate: GB 91.10%, SVM 56.60%. Severe: GB 94.40%, SVM 58.70%.	Normal: GB 99.20%, SVM 94.40%. Mild: GB 99.20%, SVM 40.10%. Moderate: GB 90.20%, SVM 48.50%. Severe: GB 92.20%, SVM 49.90%.		Normal: GB 92.20%, SVM 80.10%. Mild: GB 93.30%, SVM 49.90%. Moderate: GB 89.10%, SVM 64.50%. Severe: GB 91.10%, SVM 64.50%.	Normal: GB 95.50%, SVM 87.70%. Mild: GB 96.50%, SVM 44.40%. Moderate: GB 87.80%, SVM 54.50%. Severe: GB 88.20%, SVM 55.50%.	MCI: 99.00%. Severe CI: 96.00%. Moderate CI: 94.00%.	NA

Continued on next page

Table S16 (continued)

Ref.	Accuracy	Sensitivity	Specificity	Precision	F1 score	AUC	Other
46	NA	Usual pace: 95.60%. Fast pace: 97.00%		Usual pace: 95.40%. Fast pace: 96.30%	Usual pace: 95.50% ± 2.30%. Fast pace: 96.70% ± 2.40%	NA	NA
30	90.48%	92.00%	88.24%	NA	NA	NA	NA
31	Logistic regression (LR) 73.10%, Naive Bayes (NB) 72.30%, XGBoost (XGB) 69.30%, Random Forest (RF) 72.90%.	NA	NA	NA	NA	LR 79.70%, NB 79.90%, XGB 73.90%, RF 78.30%.	(Brier score) LR 0.189, NB 0.231, XGB 0.220, RF 0.194.
32	NA	71.00%	94.00%	NA	NA	NA	NA
33	GBDT: 66.74%, XGB: 69.27%	GBDT: 88.74%, XGB: 84.30%	NA	GBDT: 69.89%, XGB: 73.73%	GBDT: 78.20%, XGB: 78.66%	GBDT: 62.00%, XGB: 65.10%	NA

Continued on next page

Table S16 (continued)

Ref.	Accuracy	Sensitivity	Specificity	Precision	F1 score	AUC	Other
[34]	8 channels: 86.85% ± 4.97%	8 channels: 86.85% ± 6.88%	8 channels: 86.85% ± 7.81%	NA	NA	NA	NA
[47]	98.44%	96.58%	98.95%	96.11%	NA	99.7%	NA
[35]	76.50%	80.95%	85.71%				
[49]	NA	SVM: 66.00% ± 5.00%; ElasticNet: 71.00% ± 6.00%; Logistic Regression: 67.00% ± 6.00%		SVM: 45.00 ± 1.00%; ElasticNet: 44.00% ± 3.00%; Logistic Regression: 45.00% ± 3.00%	SVM: 52.00% ± 2.00%; ElasticNet: 51.00% ± 4.00%; Logistic Regression: 51.00% ± 3.00%	SVM: 76.00% ±2.00%; ElasticNet: 77.00% ± 2.00%; Logistic Regression: 78.00% ± 2.00%	NA

Continued on next page

Table S16 (continued)

Ref.	Accuracy	Sensitivity	Specificity	Precision	F1 score	AUC	Other
[45]	CNN 88.00%, ViT baseline 86.30%, ViT small 84.60%, ViT small+th 88.90%	CNN 82.50%, ViT baseline 80.00%, ViT small 67.50%, ViT small+th 87.50%	NA	CNN 82.50%, ViT baseline 80.00%, ViT small 84.40%, ViT small+th 81.40%	NA	NA	(F2 score) CNN 82.50%, ViT baseline 80.00%, ViT small 70.30%, ViT small+th 86.20%
[37]	During daily life questions: LightGBM 70.40%. During cognitive tasks: LightGBM 77.80%.	During daily life questions: LightGBM 81.30%. During cognitive tasks: LightGBM 87.50%.	During daily life questions: LightGBM 54.50%. During cognitive tasks: LightGBM 63.60%.	During daily life questions: LightGBM 76.50%. During cognitive tasks: LightGBM 82.40%.	NA	NA	NA
[38]	83.80%	NA	NA	NA	NA	78.80%, bootstrap 100x: 78.70% ± 1.50%	NA

Continued on next page

Table S16 (continued)

Ref.	Accuracy	Sensitivity	Specificity	Precision	F1 score	AUC	Other
[39]	NA	NA	NA	NA	NA	Dementia/MCI vs CN: LR 76.00%. Dementia vs CN: SVM 78.00%. MCI vs CN: LR 73.00%.	(AUPRC) Dementia/MCI vs CN: LR 32.00%. Dementia vs CN: SVM 22.00%. MCI vs CN: LR 18.00%.

Continued on next page

Table S16 (continued)

Ref.	Accuracy	Sensitivity	Specificity	Precision	F1 score	AUC	Other
41	NA	NC vs MCI: DT stride length 59.50%, DT turn velocity 73.80%; NC vs Dementia: DT stride length 71.40%, DT turn velocity 85.70%	NC vs MCI: DT stride length 94.70%, DT turn velocity 84.20%; NC vs Dementia: DT stride length 97.40%, DT turn velocity 84.20%	NA	NA	NC vs MCI: DT stride length 84.40%, DT turn velocity 80.10%; NC vs Dementia: DT stride length 92.60%, DT turn velocity 92.30%	NA
44	87.00%	87.00%	86.00%	87.00%	NA	NA	NA

Table S17: Evaluation results from research applying a regression approach. References are according to the article's references list.

**Ref.**	**Other**
[36]	(R squared) Attention: 0.214; Verbal Memory: 0.383; Executive Function: 0.414
[42]	(MAE) Global Cognition - Inter-individual: subject-based + elastic net 0.59; interval-based + random forest 0.16. Intra-individual: subject-based + random forest 0.11; interval-based + xgboost 0.09

Table S18: Extracted author sentences referring to DHT impact on the ADRD screening and diagnosis. References are according to the article's references list.

Ref.	Sentences
23	"Demonstrates objective signs of stress with increased heart rate and galvanic skin response in the individuals with MCI."
24	None
25	"Sleep state variability is promising in detecting seniors with MCI"
26	"Evidence that gait as a biomarkers could be useful for indicating the early stage of AD." "Increase stride time variability could be used to distinguish aMCI patients from normal healthy elderly." "Cognitive impairment-related gait disorders occur (...) in daily life walking among the aMCI patients." "A sensor-based wearable device for gait measurement may be an alternative and convenient tool for screening cognitive impairment."
27	"Study confirms consumer actigraphy as a useful method to better evaluate and characterize sleep and behavioral symptoms in patients with neurodegenerative diseases", "May also be adequately used to study sleep and neurodegeneration from the preclinical stages of the disease, providing validates and, therefore, objective instrumental diagnostic support."
48	"These results lead us to hypothesize that sleep duration may be an important lifestyle factor associated with cortical amyloid burden and brain function." "May contribute to the development of novel evidence-based interventions for delaying cognitive impairment in older adults."

Continued on next page

Table S18 (continued)

Ref.	Sentences
[28]	"May improve our understanding of the impact of cognitive impairment on daily living and support the development of improved methods for disease management." "Highlights the importance of considering motor problems in treatment protocols for MCI and dementia, regardless of disease subtype." "Monitoring individual trajectories of change within free-living behaviors may be more clinically useful than applying thresholds of at-risk behavior." "Has demonstrated feasibility for continuously monitoring HA in different dementia disease subtypes with wearable technology." "Wearable technology can provide a novel personalized method to measure HA in cognitively impaired populations, and may be a useful objective tool for monitoring disease progression and loss of independence."
[52]	"Strongly supports that those cognitively healthy elderly with lower sleep time are potentially at greater risk of future neurodegenerative diseases based on higher t-tau and p-tau levels in the CSF." "Suggests an early association between tau and sleep independent of AB in cognitively healthy individuals." "As a sort of canary in a coal mine, sleep disturbances might be an early AD biomarker." "This is an inexpensive and scalable technology that could be incorporated to risk prediction algorithms together with other available information."
[51]	"Demonstrated the capability to passively detect cognitive impairment symptoms by monitoring daily physical activities and keystroke patterns." "Early detection of these progressions can allow for interventions that can lessen, delay, or thwart related functional impairments."

Continued on next page

Table S18 (continued)

Ref.	Sentences
46	"This study can have a great value in real practice where precise and multilevel severity classification is essential for better selections of therapeutic modalities." "The gait sequence features that consisted of time-consecutive temporal gait parameters could effectively encapsulate the distinctive gait patterns of the three groups, and the LSTM networks could precisely recognize and learn the gait pattern of each group." " The proposed approach is expected to be easily accessible by inexpert individuals under non-experimental settings." "The proposed approach can contribute to the continuous and ubiquitous health monitoring of the elderly in daily life and consequently to prevent the waste of a valuable medical resources and cost caused by the unnecessary aggravation of CI." "Can serve as one of the first stepping stones towards future studies for less professional-oriented assessment of health risks in the elderly under non-experimental settings based on daily living activities."
29	"Body temperature measures could be combined with other predictors of cognitive performance and integrated into multi-parameter models for the prediction of cognitive decline in older adults." "Rib cage measurement more favorable for future applications in wearable monitoring devices." "Skin temperature assessed in single-point laboratory measurements correlate with both general cognitive performance and with various specific domains of cognitive performance." "Appears promising to include body temperature sensors into multi-parameter wearable systems for the remote and continuous monitoring of the older population's neurocognitive health."
30	"The experimental results show that when subjects walking, data from the upper body contribute to distinguish MCI/AD."
31	"Obvious that the proposed reading features contain important information for (early) detection of MCI." "Suggests potential for practical applicability of a clinical decision support system based on a quick reading test."
32	"Potential utility of real-world navigation patterns as an ecologically valid behavioral marker of AD"

Continued on next page

Table S18 (continued)

Ref.	Sentences
[33]	"Machine learning is a very important assistive technology for continuous monitoring and early warning of MCI, can further improve the triage decision-making ability of health care providers to identify people at high risk of MCI among the community elderly."
[34]	"Demonstrated the possibility of precise machine learning-based diagnosis of MCI with a limited number of EEG electrodes." "The proposed optimal electrode configurations showed statistically higher accuracies than the electrode configurations of commercial wearable EEG devices, albeit with a smaller number of electrodes."
[47]	"Useful as a study to determine whether it can be used as a monitoring tool for early diagnosis of dementia through the classification ACC of dementia risk group and normal group through the correlation between cognitive function and dementia related risk factors that can be collected from wearable device." "Is a non-invasive and easy-to-wear device by the elderly, so it is considered useful as a dementia risk monitoring tool."
[35]	"Real-time measures of HRV could be used as an early indicator of cognitive decline in individuals with MCI." "Using biosensors to measure HRV can be relatively reliably to distinguish cognitively normal healthy controls from MCI patients."
[49]	"These machine learning models are useful for prescreening to detect amyloid positivity and can deduce costs and the number of unnecessary invasive lumbar puncture and amyloid PET in the clinical trials on AD or clinical settings."
[36]	"Continuous monitoring of patients with accelerometers and gyroscopes (...) can be adapted to monitor the functional status in Alzheimer's disease."
[45]	None

Continued on next page

Table S18 (continued)

Ref.	Sentences
37	"Demonstrate that deficits in everyday functioning can be detected from acoustic features in voice data collected during both cognitive tasks and daily conversations." "Machine learning analysis showed that the smartwatch-derived acoustic features could differentiate individuals with high and low ECog scores with higher accuracy than the neuropsychological tests for screening cognitive impairment." "Common acoustic features can be robustly used for detecting deficits in everyday functioning from different types of voice data." "The proposed objective method may offer a complementary (or possibly alternative) approach to help improve the detection accuracy for subtle changes in everyday functioning, as well as the prognostic accuracy for related diseases." "Smartwatch-based assessment using voice data from daily conversation may enable passive, continuous monitoring for deficits in everyday functioning."
38	"Our model showed good diagnostic performance for CD, similar to the model using the MMSE, which is most widely used for screening CD in both clinical and research settings." "Compared with the MMSE-based model, our gait-based model showed a higher sensitivity but showed a lower specificity." "Gait analysis using a wearable inertial sensor can be easily and repeatedly self-administered at home and is robust to socioeducational influences and learning effects." "Advantageous for both screening and monitoring CDs and for capturing real-life functions." "Can be easily packaged into a mobile application in the future." "Possible to use our model more cheaply and widely available." "It may help to make the screening and monitoring of CD easier, wider, and cheaper for older adults."
39	"Features derived from sleep EEG can be used to discriminate DEM, MCI, and CN groups." "Results from our linear models revealed differences in feature change with age across Dem, MCI, and CN groups." "Shows that brain activity during sleep has the potential to detect features associated with dementia and contains information that can help inform individual-level clinical decision-making." "There is thus promise for clinical translation using home-based wearable EEG to routinely screen individuals for dementia and provide individual-level neuropathological estimates for diagnosis and monitoring of disease progression."

Continued on next page

Table S18 (continued)

Ref.	Sentences
40	None
41	"Playing an increasingly important role in the early screening of neurodegenerative diseases", "These significant findings highlight the potential for intelligent wearable devices to be utilized in the future of screening individuals with MCI and dementia", "Validates its potential as a novel gait marker for cognitive impairment"
50	"Together, these data suggest objective measures of 24-h activity could be used as community-based biomarkers of neurodegeneration risk.", "Accelerometer-derived metrics are an affordable and scalable monitoring system that could be used (...) to evaluate elevated risk of PD, AD, and accelerated cognitive decline in all older adults.", "24-h rhythm integrity as assessed by seven days of wrist actigraphy can serve as a prospective marker of incident AD and PD risk as well as cognitive decline"
42	"Body of evidence regarding associations between HRV measures and cognitive function in individuals with MCI." "Physiological features have potential to be used for passive assessment of cognitive functions using wearable sensors in real time and in ambulatory settings." Such continuous monitoring "would allow a better understanding of the response to treatment and the delivery of more effective personalized therapies."
43	"As an integral part of everyday human life, it is appropriate to evaluate sleep as an important modifiable risk factor for AD"

Continued on next page

Table S18 (continued)

Ref.	Sentences
[44]	"Despite processing significantly less data compared to the traditional CNN, CLADSI achieves a comparable success date." "Underscores the efficiency of CLADSI in delivering similar results while utilizing a smaller dataset." "One of the key advantages of the proposed method it its ability to continuously learn and adapt to new data over time, thus enhancing adaptability." "This method continuously self-adjusts based on new data, allowing it to adapt dynamically to changes in a patient's condition or surroundings." "It enables early detection and monitoring of gait disturbances, which can signal the onset of AD." "The ability to update the model autonomously without human intervention boosts the efficiency and scalability of medical monitoring systems." "Provide valuable insights into the application of continual learning algorithms with motion sensor data for AD identification." "Enabling more targeted and timely interventions to improve patient outcomes." "Moreover, the approach leverages widely accessible sensors such as smartphone accelerometers, meaning it can be easily integrated into existing healthcare systems."
